# Clinical and genetic determinants of the fatty liver–coagulation balance interplay in individuals with metabolic dysfunction

**DOI:** 10.1016/j.jhepr.2022.100598

**Published:** 2022-09-25

**Authors:** Luca Valenti, Armando Tripodi, Vincenzo La Mura, Serena Pelusi, Cristiana Bianco, Erica Scalambrino, Sara Margarita, Francesco Malvestiti, Luisa Ronzoni, Marigrazia Clerici, Roberta D’Ambrosio, Mirella Fraquelli, Rossana Carpani, Daniele Prati, Flora Peyvandi

**Affiliations:** 1 Fondazione IRCCS Ca’ Granda Ospedale Maggiore Policlinico, Precision Medicine Lab, Biological Resource Center, Department of Transfusion Medicine, Milan, Italy; 2 Università Degli Studi di Milano, Department of Pathophysiology and Transplantation, Milan, Italy; 3 Fondazione IRCCS Ca’ Granda Ospedale Maggiore Policlinico, Angelo Bianchi Bonomi Hemophilia and Thrombosis Center, Milan, Italy; 4 Fondazione IRCCS Ca’ Granda Ospedale Maggiore Policlinico, Gastroenterology and Hepatology, Milan, Italy; 5 Fondazione IRCCS Ca’ Granda Ospedale Maggiore Policlinico, Gastroenterology and Endoscopy, Milan, Italy

**Keywords:** NAFLD, PNPLA3, ABO blood group, von Willebrand factor, Liver fibrosis, ALT, alanine aminotransferase, AST, aspartate aminotransferase, CAP, continuous attenuation parameter, CRP, C-reactive protein, F8, factor VIII, FIB-4, Fibrosis-4, FNI, Fibrosis NASH index, FVL, factor V Leiden, GGT, gamma glutamyl transferase, GLM, generalised linear model, HOMA-IR, Homeostatic Model Assessment for Insulin Resistance, LSM, liver stiffness measurement, NAFLD, non-alcoholic fatty liver disease, NASH, non-alcoholic steatohepatitis, PC, protein C, Pro-C3, N-terminal pro-peptide of collagen-3, PRS-HFC, polygenic risk score - hepatic fat content, PRS-5, polygenic risk cosre-5, vWF, von Willebrand factor

## Abstract

**Background & Aims:**

The aim of this study was to examine the determinants of the interplay between liver damage and the coagulation balance in individuals at risk of non-alcoholic fatty liver disease (NAFLD).

**Methods:**

We considered 581 healthy participants with ≥3 metabolic alterations undergoing clinical and genomic evaluation, measurement of liver stiffness (LSM) and controlled attenuation parameter (CAP) by Fibroscan, Pro-C3, coagulation balance (von Willebrand factor [vWF], factor VIII/protein C ratio [F8/PC] as the main outcome, D-dimer as marker of coagulation/fibrinolysis activation).

**Results:**

Liver fibrosis indices (both Fibrosis-4 [FIB-4] and liver stiffness measurement [LSM]), but not liver fat (CAP), were independently associated with higher F8/PC ratio (*p* <0.01), triggering D-dimer formation (*p* = 2E-21). In keeping with a causal role of liver damage in determining a procoagulant status, the main fatty liver inherited risk variant *PNPLA3* p.I148M was independently associated with the F8/PC ratio (*p* = 0.048). *Vice versa*, the main determinant of the coagulation balance was *ABO* locus variation (*p* = 1E-16), through the impact on vWF (*p* = 8E-26). Both rs687289 *ABO* and factor V Leiden were independently associated with higher Pro-C3 (*p* <0.025), with the effect of *ABO* being mediated by the impact on vWF (*p* = 5E-10 for association with Pro-C3). Mendelian randomisation analysis was consistent with a causal association of procoagulant imbalance with heightened fibrogenesis (*p* = 0.001 at robust MR-Egger for Pro-C3), but not with fibrosis (for LSM; *p* = not significant).

**Conclusions:**

In individuals with metabolic dysfunction, liver damage severity and possibly the *PNPLA3* p.I148M variant were associated with procoagulant status. *Vice versa*, evaluation of inherited variants in *ABO* and other genes influencing coagulation was consistent with a causal role of procoagulant imbalance in activation of early stages of fibrogenesis.

**Lay summary:**

In individuals with metabolic alterations at risk of metabolic fatty liver disease, there is a tendency toward heightened blood coagulation (clotting), but the cause and the impact on the progression of liver disease remain unclear. Here we show that liver damage severity and metabolic alterations, but not hepatic fat, are mainly responsible for heightened coagulation in patients with metabolic fatty liver disease. By using genetic approaches, we showed that hepatic inflammation due to lipotoxicity may favour heightened coagulation, which in turn can trigger liver fibrosis, igniting a vicious cycle that leads to progressive liver disease.

## Highlights

• There is a complex interplay between liver damage and the coagulation balance in individuals at risk of non-alcoholic fatty liver disease.
• Non-invasive indices and genetic determinants of liver damage, but not fat, were associated with a procoagulant imbalance.
• *Vice versa*, genetic predisposition to hypercoagulability was associated with fibrogenesis.
• In individuals with metabolic dysfunction, liver damage appears to promote coagulation, which in turn can activate fibrogenesis.

## Graphical abstract

**Figure:**
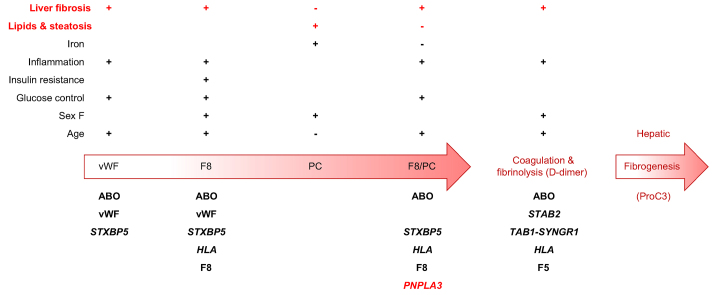

## Introduction

The liver plays a major role in the synthesis of coagulation factors and regulation of the haemostatic balance. In patients with severe liver disease, the development of cirrhosis, portal hypertension, and liver failure determines alterations in the coagulation balance hampering homeostasis maintenance and thereby leading to increased risk of both thrombotic and haemorrhagic events.^1^^,^^2^ Furthermore, it has been proposed that activation of coagulation participates actively to liver disease progression through the promotion of fibrogenesis, liver vascular occlusion, and parenchymal extinction.^3^

Non-alcoholic fatty liver disease (NAFLD), which is most frequently associated with metabolic dysfunction, is the most common cause of liver damage in the population. NAFLD has been linked with increased susceptibility to develop both liver-related and thrombotic events, the latter being the leading cause of morbidity and mortality in affected individuals.^4^ Based on cross-sectional case-control studies, we and others reported that NAFLD may tip the coagulation balance towards thrombosis more than other liver conditions,^5^ but evidence is still controversial.^6^^,^^7^

Liver fibrosis severity, the main determinant of liver-related outcomes,^8^ was linked with higher circulating levels of factor VIII (F8) paralleled by reduction in protein C (PC) activity, resulting in an enhanced F8/PC ratio. F8 is one of the most potent procoagulants in generating thrombin and PC is one of the most potent naturally occurring anticoagulants in thrombin downregulation. In addition, PC is the physiological inhibitor to F8. Heightened F8/PC ratio is also associated with high thrombin generation. Accordingly, the F8/PC ratio can be considered as an index of procoagulant imbalance *in vitro*.^5^^,^^9^ vWF is a multimeric adhesive protein, which allows platelets to adhere at the site of vessel wall injury and is also the carrier protein for F8. However, the detailed molecular mechanism underlying the association of progressive fibrosing NAFLD with the procoagulant imbalance is still under definition.^3^ Furthermore, systematic assessment of the regulation of the coagulation balance in well-characterised, unselected cohorts of individuals with metabolic risk factors, and of the causal relationship with liver damage, is still lacking.^7^

The aim of this study was to examine the determinants of the interplay (causal association) between liver damage and the coagulation balance in individuals at risk of NAFLD. This required to evaluate: (a) the clinical, metabolic, and genetic determinants of the regulation of the coagulation balance in individuals with metabolic dysfunction: this study was conducted in a prospectively enrolled cohort of 581 consecutive apparently healthy individuals without previous cardiovascular events undergoing an extensive clinical and genetic evaluation^10^; (b) to focus on the role of liver damage analysing separately non-invasively assessed hepatic fat accumulation and inflammation/fibrosis; (c) finally, to gain insight into the mechanisms underpinning the association between NAFLD and coagulation alterations, the interplay between liver disease and procoagulant phenotype was examined by a bidirectional Mendelian randomisation approach.^11^

## Patients and methods

### Study cohort

The study was conducted in participants of the Liver-Bible cohort, up to July 2021, for whom evaluation of the coagulation balance was available. These were all consecutive individuals included in the cohort, except for short periods during April–May and October 2020–February 2021, when collection of samples was interrupted to avoid potential confounding attributable to the possible impact of asymptomatic SARS-CoV-2 infection on the coagulation balance. Part of this cohort has previously been described.^10^ The Liver-Bible cohort included apparently healthy blood donors, aged 40–65 years, who were selected for a comprehensive liver disease, metabolic, and cardiovascular screening owing to the presence of at least 3 criteria of altered metabolic regulation, being overweight (BMI >25 kg/m^2^), arterial hypertension (blood pressure >133/85 mmHg or use of medication), hyperglycaemia (>100 mg/dl or use of medication), low HDL cholesterol (<45/55 mg/dl in males/females), and increased triglycerides (>150 mg/dl).^12^ They were all negative for markers of HBV and HCV infection and none reported use of alcohol ≥60/40 g/day in Males/Females.

All underwent evaluation of BMI, abdominal circumference, glucose and lipid levels, insulin, HbA1c, alanine aminotransferase (ALT), aspartate aminotransferase (AST), gamma glutamyl transferase (GGT), levels of alcohol intake (drinks/weeks), sweetened beverages (drinks/week) and moderate-intense physical activity (hours/week). Diabetes was diagnosed as HbA1c ≥49 mM.

Participants underwent Fibroscan for non-invasive evaluation of liver stiffness measurement (LSM) and continuous attenuation parameter (CAP). CAP ≥275 dB/m (despite limited sensitivity^13^) was considered consistent with the presence of fatty liver disease, while Fibrosis-4 (FIB-4) >1.3, followed by LSM ≥8 kPa were considered suggestive of advanced fibrosis, respectively, according to guidelines.^14^ Besides the FIB-4 score and LSM, we considered the Fibrosis non-alcoholic steatohepatitis (NASH) index (FNI) score to estimate liver damage severity.^15^ The choice to use multiple non-invasive predictors was guided by (a) the recommendation to use FIB-4 followed by LSM and an additional biomarker in clinical practice^14^; (b) the lack of possibility to assess the invasive gold standard (liver biopsy) in the majority of patients where it was not indicated. Fibrogenesis was assessed by measurement of the N-terminal pro-peptide of collagen-3 (Pro-C3) neo-epitope, a marker of active fibrogenesis, which showed the best correlation with fibrosis in patients with NAFLD and metabolic risk factors.^16^ Pro-C3 was measured by ELISA (BioTechne, Milan, Italy). Evaluation of coagulation factors, genotyping, and imputation are described in the Supplementary material. The main outcome we used to assess the regulation of coagulation balance was the F8/PC ratio.

The study was approved by the Ethical committee of the Fondazione IRCCS Ca’ Granda, and each participant signed a written informed consent (ID 1650, revision June 23, 2020). The clinical features of these individuals stratified by the presence of NAFLD (CAP ≥275 dB/m) are reported in Table 1.

**Table 1:** **Clinical features of the 581 participants in the LIVER-BIBLE-2021 cohort who underwent evaluation of the coagulation balance and genomic characterisation, stratified by the presence of NAFLD (as detected by CAP ≥275 dB/m)**.

	Fatty liver (CAP ≥ or <275 dB/m)			
	**Yes**	**No**	***p* value**∗	***p* value**†
**n =**	296 (50.3)	285 (49.7)		
Age, years	53.9 (6.3)	53.4 (6.4)	0.36	0.36
Sex, female	39 (13.2)	43 (15.1)	0.52	0.52
Ethnicity, European	284 (96.2)	274 (96)	0.99	0.99
BMI, kg/m^2^	29.5 (3.1)	27.6 (2.6)	**1.00E-13**	**1.00E-13**
Obesity, yes	102 (34.6)	30 (10.5)	**3.00E-12**	**4.00E-10**
Abdominal circumference, cm	105.8 (9.2)	100.3 (7.4)	**2.00E-13**	**2.00E-13**
Glucose	97.2 (12.8)	95.4 (13.6)	0.11	0.16
Insulin, mU/L	16.4 (9.8)	13.2 (7.9)	**2.00E-05**	**2.00E-05**
HOMA-IR, units	3.9 (2.5)	3.1 (2)	**2.00E-05**	**3.00E-05**
HbA1c, mM	35.9 (4.7)	35.1 (3.7)	**0.023**	**0.027**
Diabetes, yes	16 (5.5)	8 (2.8)	0.13	0.13
Hypertension, yes	210 (71.2)	183 (64.2)	0.08	0.13
LDL, mg/dl	121.7 (28.2)	123.9 (29.9)	0.33	0.36
HDL, mg/dl	44 (8.7)	45.9 (10.9)	**0.019**	**0.014**
Triglycerides, mg/dl	119 (85-161)	110 (79-160)	0.26	0.24
ALT, IU/L	25 (28-34)	22 (19-28)	**0.0003**	**0.0002**
AST, IU/L	23 (20-26)	22 (19-26)	**0.045**	**0.044**
GGT, IU/L	24 (18-33)	22 (16-31)	0.083	0.094
Ferritin	75 (39-125)	73 (43-121)	0.25	0.23
CAP, dB/m	244 (22.5)	309.3 (25.7)	0	0
FIB-4, score	1.1 (0.3)	1.1 (0.4)	0.79	0.41
Pro-C3, ng/ml	7.9 (6.6-9.7)	7.5 (6.2-9.3)	0.17	0.18
LSM, kPa	5.3 (1.3)	4.7 (1.3)	**1.00E-06**	**1.00E-06**
vWF:Ag, U/dl	125.6 (39.9)	124.8 (37.8)	0.79	0.92
F8, U/dl	96.5 (26.3)	95.9 (26.7)	0.79	0.89
PC, U/dl	113.1 (18.4)	110.8 (18.7)	0.12	0.08
F8/PC, ratio	0.87 (0.27)	0.89 (0.29)	0.48	0.33
D-Dimer, ng/ml	263 (188-366)	264 (177-387)	0.91	0.82

ALT, alanine aminotransferase; AST, aspartate aminotransferase; CAP, continuous attenuation parameter; F8, factor VIII; FIB-4, Fibrosis-4; GGT, gamma glutamyl transferase; GLM, generalised linear model; HOMA-IR, Homeostatic Model Assessment for Insulin Resistance; LSM, liver stiffness measurement; NAFLD, non-alcoholic fatty liver disease; PC, protein C; vWF, von Willebrand factor.

∗ At GLM (unadjusted).

† At GLM (adjusted for age, sex, ethnicity). Values in bold denote statistical significance. Continuous variables are expressed as mean (SD) or median (IQR) and categorical variables as n (%).

### Statistical analysis

For descriptive statistics, categorical variables are shown as number and proportion. Continuous variables are shown as mean and SD or median and IQR, as appropriate. Variables that were not normally distributed (*e.g.* D-dimer) were log-transformed before entering the analyses.

Observational associations were performed by fitting data to generalised linear models (GLMs). GLMs were adjusted for age, sex, ethnicity, and clinical factors significantly associated at univariate analysis (selecting most robust determinant in each category to avoid collinearity). In the models, we introduced genetic risk variants associated (with *p* <0.1) with the trait of interest at univariate analysis. Interaction terms were introduced to check for synergic effect between risk factors, and interaction with sex was used to model the impact of X chromosome genetic variants.

To assess the reciprocal causal relationship between NAFLD and alterations in the coagulation balance, we exploited Mendelian randomisation,^17^^,^^18^ as described in detail in the Supplementary material^17^^,^[19], [20], [21], [22], [23] by the MendelianRandomization R package (R Foundation for Statistical Computing, Vienna, Austria).^24^

Statistical analysis was carried out using the JMP Pro 16.0 Statistical Analysis Software (SAS Institute, Cary, NC), and R statistical analysis software version 4.1.3 (R Foundation for Statistical Computing). Values of *p* <0.05 (2-tailed) were considered significant.

## Results

### Study cohort

The clinical features of participants in the study, stratified by the presence of NAFLD as non-invasively estimated by CAP measurement are shown in Table 1. NAFLD was detected in about half (50.3%) of participants, and expectedly it was linked with measures of adiposity (obesity, BMI, abdominal circumference), insulin resistance (fasting insulin, Homeostatic Model Assessment for Insulin Resistance [HOMA-IR] index, HbA1c, low HDL), aminotransferases and liver fibrosis (as estimated by LSM). When considering CAP as a continuous variable, it was associated with increased LSM (0.008 ± 0.001, *p* = 1E-08).

We did not detect any significant association of NAFLD with the coagulation parameters nor with the coagulation balance, although there was a non-significant trend for lower PC levels in individuals with as compared with those without fatty liver (adjusted *p* = 0.08).

### Coagulation balance determinants

The determinants of the coagulation balance in the study cohort are shown in Table 2. Older age was associated with a progressive shift of the coagulation balance towards hypercoagulability, as detected by higher vWF and F8 levels and lower PC levels, resulting in a strong impact on F8/PC ratio. Sex did not affect vWF levels. Female sex was independently associated with higher F8, but also with higher PC levels, resulting in an even F8/PC balance.

**Table 2:** **Independent determinants of the coagulation balance and activation (vWF, F8, PC, F8/PC ratio) in the 581 individuals of the LIVER-BIBLE-2021 cohort with coagulation and genetic data available**.

A	vWF:Ag, U/dl						F8, U/dl					
	**Beta**∗	**SE**∗	***p* value**∗	**Beta**†	**SE**†	***p* value**†	**Beta**∗	**SE**∗	***p* value**∗	**Beta**†	**SE**†	***p* value**†
Age, years	1.36	0.23	**7.00E-09**	1.03	0.25	**4.00E-05**	0.97	0.16	**3.00E-09**	0.56	0.18	**0.0017**
Sex, female	2.17	2.23	0.33	3.73	2.03	0.07	3.32	1.6	**0.033**	4.45	1.45	**0.0022**
Ethnicity, European	0.76	4.11	0.86	3.41	3.74	0.36	3.35	2.88	0.24	0.57	2.68	0.83
BMI, kg/m^2^	-0.13	0.5	0.79				-0.35	0.35	0.2			
Obesity, yes	-0.08	0.81	0.97				0.81	1.27	0.52			
Abdominal circumference, cm	0.07	0.18	0.7				-0.03	0.12	0.82			
Glucose	0.28	0.11	**0.0098**				0.29	0.07	**0.0001**			
Insulin, mU/L	0.31	0.17	0.063				0.3	0.12	**0.011**	0.26	0.11	**0.025**
HOMA-IR, units	1.6	0.65	**0.014**				1.53	0.46	**0.0008**			
HbA1c, mM	0.88	0.36	**0.015**	0.83	0.34	**0.016**	1.07	0.25	**2.00E-05**	0.91	0.24	**0.0002**
Diabetes, yes	10.38	3.72	**0.0054**				8.55	2.6	**0.001**			
Hypertension, yes	0.28	1.66	0.87				0.88	1.16	0.45			
LDL, mg/dl	-0.08	0.05	0.14				-0.04	0.04	0.32			
HDL, mg/dl	0.29	0.15	0.055				0.21	0.11	0.054			
Triglycerides, log mg/dl	-4.92	3.34	0.14				-2.39	2.34	0.31			
ALT, log IU/L	-3.84	3.84	0.32				0.63	2.65	0.81			
AST, log IU/L	-0.04	5.83	0.99				4.33	3.98	0.28			
GGT, log IU/L	-1.89	2.83	0.5				-0.57	1.98	0.77			
CRP, mg/dl‡	19.62	5.98	**0.001**				14.24	4.24	**0.0008**			
Ferritin, log ng/ml	-3.27	1.81	0.07				-5.01	1.26	**7.00E-05**	-1.47	1.24	0.22
Platelets, 10^3^/mm^3^	-0.06	0.03	0.063				-0.03	0.02	0.25			
CAP, dB/M	0.04	0.04	0.25				0.02	0.03	0.34			
FIB-4, score	16.3	4.26	**0.0001**	10.03	4.32	**0.02**	11.07	2.97	**0.0002**	8.92	3.06	**0.0036**
LSM, kPa	1.24	1.14	0.28				0.66	0.8	0.42			
PRS-HFC, score	4.77	8.1	0.56				9.65	5.66	0.088			
PRS-5, score	4.62	8.1	0.57				9.63	5.66	0.089			
*PNPLA3* p.I148M, alleles	1.7	2.5	0.5				3.05	1.76	0.081			
*TM6SF2*, p.E167K alleles	-1.78	2.54	0.75				1.64	4.02	0.68			
PRS-F8, score	152.57	14.24	**9.00E-27**				92.45	10.2	**1.00E-19**			
rs7135039 *vWF*, T alleles	4.17	2.38	0.079	6.49	2.11	**0.0021**	2.96	1.67	0.076	4.4	1.5	**0.0033**
rs4981022 *STAB2*, A alleles	-0.97	2.49	0.73				-0.38	1.75	0.82			
rs137631 *TAB1-SYNGR1*, C alleles	4.03	3.5	0.25				-1.84	2.46	0.45			
rs548630 *FCHO2-TMEM171-TNPO1*, C alleles	0.41	2.27	0.86				1.06	1.59	0.51			
rs9271597 *HLA,* A alleles	-2.13	2.36	0.37				-3.17	1.66	0.056	-3.96	1.47	**0.0073**
rs9399599 *STXBP5,* T alleles	6.82	2.28	**0.0028**	6.6	2.03	**0.0011**	4.01	1.6	**0.012**	3.13	1.44	**0.0298**
rs7816579 *SCARA5,* G alleles	2.22	2.59	0.39				1.95	1.81	0.28			
rs10102164 *SOX17-RP1,* A alleles	-0.93	3.19	0.77				-0.8	2.24	0.72			
rs687289 *ABO,* G alleles	-22.6	6.15	**8.00E-26**	-23.6	2.07	**4.00E-30**	-12.76	1.55	**2.00E-16**	-13.92	1.48	**4.00E-21**
rs150926226 *TMLHE-F8,* C alleles	1.43	2.57	0.58				4.89	1.79	**0.0064**	6.13	2.72	**0.027**


B	PC, U/dl						F8/PC, ratio					
	**Beta**∗	**SE**∗	***p* value**∗	**Beta**†	**SE**†	***p* value**†	**Beta**∗	**SE**∗	***p* value**∗	**Beta**†	**SE**†	***p* value**†
Age, years	-0.32	0.11	**0.005**	-0.21	0.12	0.087	0.01	0	**5.00E-11**	0.006	0.002	**0.0006**
Sex, female	3.79	1.06	**0.0004**	3.18	1.07	**0.0031**	0	0.02	1	-0.017	0.015	0.28
Ethnicity, European	0.73	1.97	0.71	3.99	1.87	**0.033**	0.03	0.03	0.35	-0.02	0.03	0.47
BMI, kg/m^2^	-0.1	0.24	0.69				0	0	0.34			
Obesity, yes	-0.75	0.87	0.39				0.01	0.01	0.47			
Abdominal circumference, cm	-0.05	0.08	0.57				0	0	0.78			
Glucose	0	0.05	0.96				0.003	0.001	**0.0006**			
Insulin, mU/L	0.08	0.08	0.32				0.002	0.001	0.16			
HOMA-IR, units	0.34	0.21	0.28				0.01	0	**0.021**			
HbA1c, mM	0.34	0.17	0.051	0.22	0.17	0.19	0.006	0.002	**0.0132**			
Diabetes, yes	-0.91	0.8	0.61				0.09	0.03	**0.0008**	0.072	0.026	**0.0059**
Hypertension, yes	0.93	0.77	0.24				-0.02	0.01	0.21			
LDL, mg/dl	0.11	0.02	**2.00E-05**	0.09	0.02	**0.0001**	-0.001	0	**0.001**	0	0	0.25
HDL, mg/dl	0.08	0.07	0.29				0.002	0.001	0.091			
Triglycerides, log mg/dl	9.8	1.6	**3.00E-10**	9.7	1.57	**6.00E-10**	-0.1	0.02	**3.00E-05**	-0.1	0.02	**3.00E-05**
ALT, log IU/L	3.94	1.84	**0.032**				-0.02	0.03	0.38			
AST, log IU/L	-0.52	2.79	0.85				0.04	0.04	0.35			
GGT, log IU/L	3.1	1.35	**0.022**	1.11	1.37	0.42	-0.02	0.02	0.26			
CRP, mg/dl‡	3.46	2.78	0.22				0.08	0.04	**0.0485**			
Ferritin, log ng/ml	2.7	0.87	**0.0019**	2.18	0.85	**0.011**	-0.07	0.01	**2.00E-07**	-0.038	0.013	**0.035**
Platelets, 10^3^/mm^3^	0.08	0.01	**4.00E-08**	0.06	0.02	**0.0024**	-0.001	0	**0.0006**			
CAP, dB/M	0.04	0.02	**0.033**	0.04	0.01	**0.01**	0	0	0.6			
FIB-4, score	-9.99	2.02	**7.00E-07**	-1.6	2.66	0.54	0.17	0.03	**2.00E-08**	0.11	0.33	**0.0004**
LSM, kPa	-1.38	0.55	**0.013**	-1.48	0.54	**0.0063**	0.02	0.01	**0.0048**	0.022	0.008	**0.0052**
PRS-HFC, score	0.45	3.86	0.91				0.06	0.06	0.27			
PRS-5, score	0.09	3.86	0.98				0.07	0.06	0.24			
*PNPLA3* p.I148M, alleles	-0.89	1.19	0.46				0.031	0.018	0.09	0.032	0.016	**0.0438**
*TM6SF2*, p.E167K alleles	0.35	2.74	0.9				0.01	0.04	0.89			
PRS-F8, score	-2.77	7.43	0.71				0.88	0.11	**2.00E-16**			
rs7135039 *vWF*, T alleles	0.74	1.14	0.51				0.024	0.017	0.16			
rs4981022 *STAB2*, A alleles	-1.83	1.18	0.12				0.017	0.018	0.33			
rs137631 *TAB1-SYNGR1*, C alleles	0.27	1.67	0.87				-0.018	0.025	0.47			
rs548630 *FCHO2-TMEM171-TNPO1*, C alleles	0.31	1.08	0.78				0.013	0.016	0.42			
rs9271597 *HLA,* A alleles	0.84	1.19	0.45				-0.038	0.017	**0.028**	-0.026	0.015	0.089
rs9399599 *STXBP5,* T alleles	0.68	1.1	0.53				0.031	0.017	0.068	0.026	0.016	0.077
rs7816579 *SCARA5,* G alleles	-0.57	1.23	0.64				0.022	0.018	0.25			
rs10102164 *SOX17-RP1,* A alleles	-2.43	1.52	0.11				0.01	0.02	0.74			
rs687289 *ABO,* G alleles	0.33	1.12	0.77				-0.119	0.016	**3.00E-13**	-0.122	0.015	**6.00E-16**
rs150926226 *TMLHE-F8,* C alleles	1.28	1.28	0.3				0.036	0.019	0.05	0.018	0.016	0.27

Values in bold denote statistical signifincance. ALT, alanine aminotransferase; AST, aspartate aminotransferase; CAP, continuous attenuation parameter; CRP, C-reactive protein; F8, factor VIII; FIB-4, Fibrosis-4; GGT, gamma glutamyl transferase; GLM, generalised linear model; HOMA-IR, Homeostatic Model Assessment for Insulin Resistance; LSM, liver stiffness measurement; PC, protein C; vWF, von Willebrand factor.

∗ At GLM (unadjusted).

† At GLM adjusted for reported variables.

‡ Available in 419.

Glucose control, as reflected by HbA1c, glucose levels, and diabetes diagnosis, was independently associated with higher vWF, resulting in a parallel increase in F8 and F8/PC ratio, the latter more evident in the presence of diabetes. However, it did not directly influence PC levels. Interestingly, the component related to insulin resistance, as detected by fasting insulin levels, was independently associated with F8, but not with vWF nor with F8/PC. HbA1c was independently associated with vWF levels both in patients with (n = 38; estimate 2.64 ± 1.22; *p* = 0.037) and without abnormal glucose metabolism (n = 543; estimate 0.73 ± 0.30; *p* = 0.017), as determined by HbA1c < or ≥42.

A similar pattern was observed for C-reactive protein (CRP) levels in the subset of the cohort where data were available, which were associated with higher vWF, F8, and F8/PC ratio, but not directly with PC. There was a tendency for an association between lower circulating ferritin with higher vWF and F8 levels, and an independent association with lower PC and higher F8/PC ratio. This trend was opposite to that of CRP, suggesting it was accounted for by depleted iron stores and not by inflammation.

Concerning lipid metabolism, increased circulating lipid levels were independently associated with higher PC, resulting in a decreased F8/PC ratio. The same trend, with independent association with higher PC levels, were observed for indices of hepatic fat accumulation, especially for the CAP score.

**Fig. 1:**
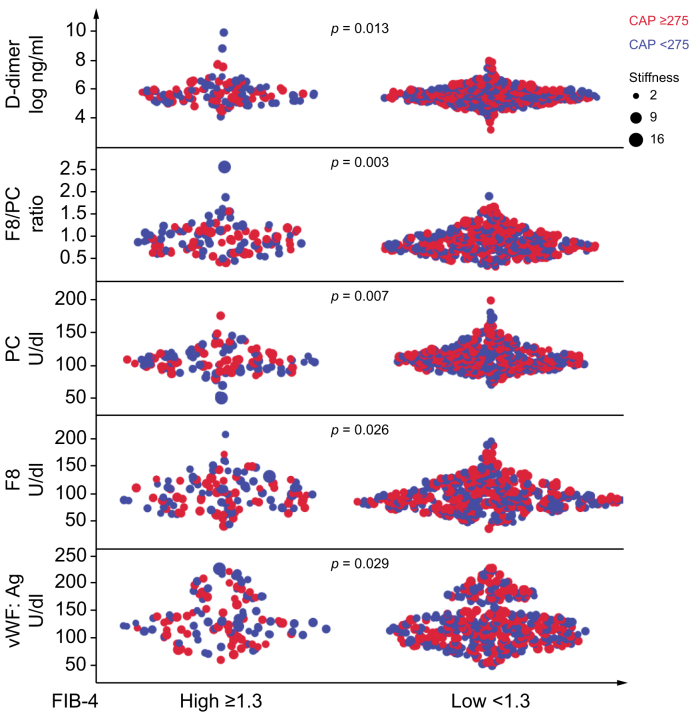
Impact of liver damage. As estimated as altered FIB-4 index (≥1.3) on the coagulation parameters considered in the 581 participants of the LIVER-BIBLE-2021 cohort. The impact of CAP (≥ or < 275) is coded by colour, whereas liver stiffness is represented by dot size. Unadjusted *p* values are reported. The *p* values were calculated with a multivariable GLM. CAP, continuous attenuation parameter; F8, factor VIII; FIB-4, Fibrosis-4; GLM, generalised linear model; PC, protein C; vWF, von Willebrand factor.

However, non-invasive predictors of liver fibrosis severity were consistently independently associated with a procoagulant imbalance. Indeed, the FIB-4 score was associated with higher vWF and F8 levels, whereas both FIB-4 and LSM were associated with lower PC and higher F8/PC ratio. In the subset of participants for whom CRP levels were available, FIB-4 remained associated with F8/PC ratio independently of CRP (estimate 0.11 ± 0.4; *p* = 0.0021; evaluated in the full model in Table 2 plus CRP levels), whereas CRP lost significance (*p* = 0.10). The impact of FIB-4 on the coagulation parameters is shown in Fig. 1.

### Contribution of genetic factors to the coagulation balance

The frequency distribution of the genetic variants analysed, for which previous associations were fully corrected, are reported in Table S1. Genetic predisposition to NAFLD and fibrosing fatty liver (polygenic risk score - hepatic fat content [PRS-HFC]/ polygenic risk score-5 [PRS-5]) did not affect the coagulation balance, but the *PNPLA3* p.I148M variant tended to be associated with higher F8 levels and was independently associated with higher F8/PC ratio independently of liver damage and of all the other covariates (*p* = 0.0438; *p* >0.087 after correction for multiplicity).

Genetic predisposition to higher F8 levels (PRS-F8) was robustly associated with vWF, F8 and the F8/PC ratio. The *ABO* locus was by far the main single genetic (and non-genetic) determinant of levels of vWF, F8, and F8/PC ratio. Variation of *VWF* was associated with vWF and F8 levels, whereas *F8* variation with F8 and F8/PC. *STXBP5* variation was also associated with vWF and F8 levels, whereas *HLA-A* variation with F8 and F8/PC (*p* <0.05 for all).

### Determinants of D-dimer levels

Levels of D-dimer were strongly associated with the F8/PC ratio (estimate 0.78 ± 0.08, *p* = 2E-21, Table 3), confirming that higher F8/PC predisposed to activation of the coagulation in this cohort.

**Table 3:** **Independent determinants of D-dimer in the 581 individuals of the LIVER-BIBLE-2021 cohort with coagulation and genetic data available**.

	D-dimer, log ng/ml					
	**Beta**∗	**SE**∗	***p* value**∗	**Beta**†	**SE**†	***p* value**†
F8/PC, ratio	0.78	0.08	**2.00E-21**			
Age, years	0.02	0.003	**3.00E-07**	0.011	0.004	**0.016**
Sex, female	0.12	0.0386	**0.0009**	0.11	0.04	**0.006**
Ethnicity, European	0.016	0.068	0.81	-0.02	0.07	0.75
BMI, kg/m^2^	0.0005	0.0008	0.54			
Obesity, yes	-0.027	0.03	0.37			
Abdominal circumference, cm	0	0.003	0.94			
Glucose	0.001	0.002	0.55			
Insulin, mU/L	0.001	0.002	0.77			
HOMA-IR, units	0.007	0.01	0.51			
HbA1c, mM	0.014	0.06	**0.017**	0.007	0.006	0.26
Diabetes, yes	0.04	0.06	0.52			
Hypertension, yes	0.008	0.027	0.78			
LDL, mg/dl	0	0.001	0.77			
HDL, mg/dl	0	0.002	0.87			
Triglycerides, log mg/dl	-0.05	0.06	0.35			
ALT, log IU/L	-0.14	0.06	**0.027**			
AST, log IU/L	-0.028	0.095	0.77			
GGT, log IU/L	-0.11	0.047	**0.018**			
CRP, mg/dl‡ˆ	0.4	0.095	**2.00E-05**			
Ferritin, log ng/ml	-0.1	0.03	**0.0008**	-0.06	0.03	0.09
Platelets, 10^3^/mm^3^	-0.001	0.001	0.051			
CAP, dB/M	-0.001	0.001	0.23			
FIB-4, score	0.29	0.07	**3.00E-05**	0.23	0.08	**0.0038**
LSM, kPa	-0.01	0.018	0.69			
PRS-HFC, score	0.045	0.137	0.74			
PRS-5, score	0.059	0.137	0.66			
*PNPLA3* p.I148M, alleles	0.024	0.04	0.58			
*TM6SF2*, p.E167K alleles	0.049	0.097	0.61			
PRS-F8, score	0.25	0.26	0.34			
rs7135039 *vWF*, T alleles	-0.01	0.04	0.74			
rs4981022 *STAB2*, A alleles	-0.11	0.04	**0.0098**	-0.1	0.04	**0.015**
rs137631 *TAB1-SYNGR1*, C alleles	0.13	0.06	**0.022**	0.12	0.06	**0.03**
rs548630 *FCHO2-TMEM171-TNPO1*, C alleles	-0.006	0.038	0.87			
rs9271597 *HLA,* A alleles	-0.08	0.04	**0.046**	-0.07	0.04	0.069
rs9399599 *STXBP5,* T alleles	-0.06	0.04	0.12			
rs7816579 *SCARA5,* G alleles	0.01	0.04	0.74			
rs10102164 *SOX17-RP1,* A alleles	-0.08	0.05	0.14			
rs687289 *ABO,* G alleles	-0.07	0.04	0.076	-0.08	0.03	**0.027**
rs150926226 *TMLHE-F8,* C alleles	0.07	0.04	0.13			
rs6025 *FVLeiden*, alleles	0.31	0.15	**0.036**	0.3	0.14	**0.033**
rs1799963 *PT20210*, alleles	0.19	0.13	0.15			

Values in bold denote statistical signifincance. ALT, alanine aminotransferase; AST, aspartate aminotransferase; CAP, continuous attenuation parameter; CRP, C-reactive protein; F8, factor VIII; FIB-4, Fibrosis-4; GGT, gamma glutamyl transferase; GLM, generalised linear model; HOMA-IR, Homeostatic Model Assessment for Insulin Resistance; LSM, liver stiffness measurement; PC, protein C; vWF, von Willebrand factor.

∗ At GLM (unadjusted).

† At GLM adjusted for reported variables.

‡ Available in 419.

When considering each single determinant separately (reported in Table 3), D-dimer levels were independently associated with older age, female sex, and higher FIB-4 score. Concerning the genetic risk factors, among the determinants of F8 D-dimer levels were associated with variation in *STAB2*, *TAB1-SYNGR1*, and *ABO*, and were also higher in carriers of factor V Leiden (FVL) (*p* <0.05 for all). However, D-dimer was not associated with genetic predisposition to NAFLD. In the subset of participants where data were available, when introduced in the model CRP levels predicted D-dimer (estimate 0.34 ± 0.10, *p* = 0.0009).

### The role of the ABO blood group

**Fig. 2:**
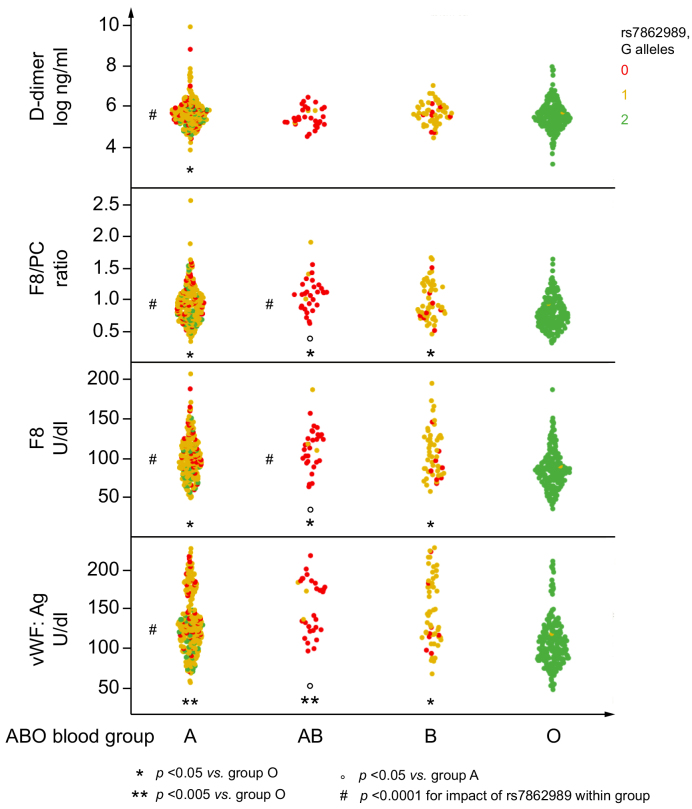
Combined impact of ABO blood group and rs687289 A>G genotype on the coagulation balance in the 581 participants in the LIVER-BIBLE-2021 cohort. The *p* values were calculated with a multivariable generalised linear model.

As the *ABO* locus was the main independent determinant of vWF levels and consequently of the whole coagulation balance (the association of rs687289 with F8, F8/PC, and D-dimer was lost after correction for vWF levels, *p* >0.5 for all), we next focused the attention on the role of ABO blood group. The impact of ABO blood group on the coagulation balance is shown in Fig. 2. Carriers of non-O blood groups had higher levels of vWF, F8, F8/ratio, and D-dimer levels as compared with group O carriers, and more so those carrying the AB group than the A group (*p* <0.05 for all). In addition, the rs786298 G allele was significantly associated with lower vWF and coagulation parameters in carriers of blood group A and AB (*p* <0.05). At multivariable analysis, the ABO blood group underestimated the impact of *ABO* locus variation on vWF and consequently on the coagulation balance, as the B blood group and rs687298 G allele contributed independently to vWF levels (Table 4, left panel). Furthermore, there was a significant interaction between the rs687298 G allele and rs8176746 T allele associated with B encoding *ABO* haplotypes in determining vWF levels (Table 4, right panel).

**Table 4:** **Combined impact of *ABO* locus variation and ABO blood group on circulating vWF:Ag concentration**.

vWF:Ag, U/dl	Estimate	SE	*p* value	Estimate	SE	*p* value
Age, years	0.99	0.25	**6.00E-05**	0.97	0.25	**7.00E-05**
Sex, female	3.82	2.04	0.061	3.77	2.03	0.063
HbA1c, nM	0.92	0.34	**0.0071**	0.89	0.34	**0.009**
FIB-4, score	9.79	4.30	**0.0227**	10.52	4.27	**0.0138**
rs9399599 *STXBP5*, T alleles	6.26	2.01	**0.0018**	6.34	2.00	**0.0015**
rs7135039 *vWF*, T alleles	6.59	2.09	**0.0016**	6.55	2.08	**0.0017**
rs687289 *ABO*, G alleles	-16.50	3.83	**1.00E-05**	-19.78	2.34	**3.00E-17**
ABO group A	-2.25	2.48	0.36			
ABO group B	10.73	3.79	**0.0047**			
ABO group AB	2.96	5.79	0.61			
ABO group O	ref					
rs8176746 *ABO*, T alleles				24.74	6.37	**0.0001**
rs8176746∗rs687289				22.89	6.59	**0.0005**

The *p* values were calculated with a multivariable GLM. Values in bold denote statistical signifincance. FIB-4, Fibrosis-4; GLM, generalised linear model; vWF, von Willebrand factor.

#### Independent determinants of liver fibrosis

The independent determinant of liver damage and fibrosis, estimated by FIB-4 score, LSM, and FNI score are presented in Table S2. The procoagulant imbalance (F8/PC ratio) was independently associated with fibrosis (*p* <10E-04 for FIB-4, *p* <0.05 for LSM and FNI). Similar data were obtained for altered FIB-4 and PNI score (*p* <0.05 for a direct association with F8/PC in the fully adjusted logistic regression model). Genetic predisposition to higher F8 levels (PRS-F8 score) was not associated with more severe fibrosis. These data confirm that F8/PC ratio is linked with liver fibrosis severity.

#### Mendelian randomisation analysis

Finally, we exploited a Mendelian randomisation approach to investigate the causality and direction of the epidemiological association between liver fibrosis and the procoagulant status. Detailed results are reported in the Supplementary material, Figs S1 and S2, and Tables S3 and S4. Results were consistent, but not conclusive, with a causal association of fibrosing NAFLD with procoagulant imbalance. *Vice versa*, although the F8/PC ratio was not causally associated with liver fibrosis, we observed a causal association of genetic predisposition to procoagulant imbalance with pro-C3 at most estimates (Table S4, right column and Fig. S2), which was confirmed with sensitivity analyses.

We then therefore looked directly at the impact of *ABO* on Pro-C3. The independent determinants of Pro-C3 levels are shown in Table 5. We confirmed that rs687289 *ABO* G alleles, associated with lower vWF and F8/PC ratio, showed a protective association with Pro-C3 (Table 5; *p* = 0.023). Importantly, we also showed that carriage of FVL favouring a procoagulant imbalance downstream of F8/PC was also independently associated with Pro-C3 (*p* = 0.021). In addition, the impact of *ABO* locus on fibrogenesis was fully mediated by modulation of vWF levels, as when circulating vWF concentration was introduced in the model it abolished the effect of rs687289 A>G and was independently associated with Pro-C3 (*p* = 5E-10).

**Table 5:** **Independent determinants of circulating Pro-C3 levels (log ng/ml) in the 581 individuals of the LIVER-BIBLE-2021 cohort with coagulation and genetic data available**.

Pro-C3, log ng/ml	Estimate	SE	*p* value	Estimate	SE	*p* value
Age, years	0.021	0.004	5.00E-07	0.014	0.004	0.00057
Sex, female	0.124	0.038	0.0011	0.090	0.037	0.016
BMI, Kg/m2	-0.001	0.009	0.89	0.002	0.009	0.84
FNI, score	0.583	0.260	0.025	0.348	0.254	0.17
rs687289 *ABO,* G alleles	-0.088	0.039	0.023	-0.001	0.040	0.98
rs6025 *FVLeiden*, alleles	0.334	0.145	0.021	0.277	0.140	0.048
vWF:Ag, U/dl				0.007	0.001	5.20E-10

The *p* values were calculated with a multivariable GLM. FNI, Fibrosing NASH Index; GLM, generalised linear model; vWF, von Willebrand factor.

## Discussion

**Fig. 3:**
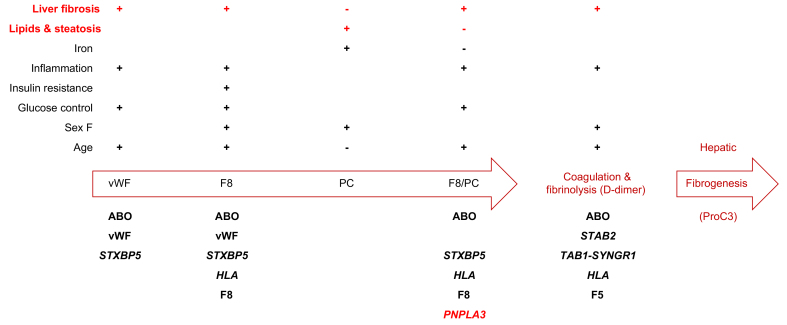
Graphical overview of the main clinical (above) and genetic (below) determinants of the coagulation parameters identified in the present study in apparently healthy individuals with metabolic risk factors. F8, factor VIII; PC, protein C; vWF, von Willebrand factor.

In this study, we first examined the clinical and genetic determinants of the coagulation balance, as assessed by the F8/PC ratio and D-dimer, in individuals with multiple metabolic alterations at high risk of NAFLD, focusing on the role of liver damage. A graphic summary of the main findings is presented in Fig. 3. We confirmed that liver inflammation and fibrosis, as assessed by non-invasively FIB-4 and LSM, were independently associated with a procoagulant shift, as a consequence of upregulation of vWF, circulating F8, and reduction of PC, resulting in increased activation of coagulation and fibrinolysis.^5^^,^^9^ Of note, FIB-4 was associated with the full spectrum of the coagulation balance, whereas LSM with reduced PC levels, suggesting that hepatic inflammation and fibrosis may selectively affect haemostasis. However, we did not observe any contribution of hepatic fat accumulation, as estimated by CAP, on the procoagulant shift typical of patients with NAFLD, after adjustment for the impact of liver damage and of metabolic risk factors. Indeed, glucose control and HbA1c were linked to higher vWF, F8, and procoagulant imbalance, whereas insulin resistance was related directly to F8 levels. In keeping, glucose control has previously been associated with circulating vWF levels.^25^^,^^26^ Conversely, liver fat content and, in line with previous findings,^27^ circulating lipids were associated with increased levels of PC. These data suggest that the procoagulant alterations typically observed in patients with NAFLD are not directly mediated by hepatic fat accumulation but may be accounted for by subclinical liver disease and by the association with impaired glucose metabolism. Similarly, liver fibrosis, but not steatosis, was associated with a procoagulant imbalance in chronic hepatitis C patients.^28^ However, we could not assess and draw conclusions on the regulation of fibrinolysis, which in a recent study was found impaired in non-diabetic patients with NAFLD,^29^ and on the role of neutrophil extracellular traps in triggering coagulation in participants with liver inflammation.^30^ Notwithstanding, the present results seem to confirm that subclinical systemic inflammation (CRP levels) impact on the coagulation balance at different levels,^31^ even in this cohort of apparently healthy and asymptomatic individuals, but most importantly showed that the association of early liver damage with procoagulant imbalance is independent of CRP. However, the association of low ferritin with reduced PC, going in the opposite direction of that of CRP and thereby likely reflecting a deficit in iron stores, seems novel and deserves further investigation.

A strength of the present study was the comprehensive evaluation of the main common genetic determinants of both NAFLD and of vWF/F8 levels, for which the epidemiological associations and risk estimates were adjusted for. In addition, we could assess the impact of these genetic factors on the coagulation balance (Fig. 3). The main inherited determinant of the coagulation balance was genetic variation at the *ABO* locus encoding for the ABO blood group. Remarkably, the effect was several-fold larger (>5-fold) of all other inherited determinants and of clinical factors as well. These data are in line with the literature indicating that *ABO* is the main inherited determinant of circulating vWF^23^ and a major determinant of the risk of thrombosis.^32^ As vWF is a substrate of the ABO protein, the mechanism is likely mediated by reduced vWF clearance in non-O blood carriers owing to qualitatively different vWF N-glycan composition (reviewed in Franchini *et al.*^32^). The impact of ABO extended to the all levels of the coagulation balance evaluated, from vWF to F8, F8/PC ratio, and activation of coagulation/fibrinolysis (D-dimer), although as expected it was entirely mediated by increased vWF levels. Another notable finding was that the impact of *ABO* variation on vWF levels was not fully accounted by ABO blood group, particularly in A+ individuals. Indeed, in the present cohort the rs687289 G allele, an expression quantitative trait locus reducing ABO protein levels,^33^ was associated with lower vWF in blood group A carriers and contributed to vWF levels independently of haplotypes encoding non-O blood groups. These data are again consistent with the notion that increased glycation reduces VWF clearance. In keeping, another independent determinant of vWF was HbA1c, a marker of abnormal glycation of circulating proteins in response to hyperglycaemia, whereas no independent impact of insulin resistance was observed. Therefore, rs687289 genotyping may add additional prognostic information on the risk of thrombosis as compared with the mere knowledge of ABO blood group. Concerning other genetic factors, we validated in the cohort the role of single determinants at each specific step, including a role for *VWF* and *F8* variation on their respective protein levels, *STXBP5* on vWF, *HLA* on F8, *STAB2*, *TAB1-SYNGR1*, and *F5* on the activation of coagulation downstream.

The observation that inheritance of the *PNPLA3* p.I48M variant, the main genetic determinant of progressive NAFLD, through action on hepatic fat, lipotoxicity, and hepatic stellate cells – fibrogenesis,^34^ was independently associated with higher F8/PC levels is consistent with the hypothesis that liver damage plays a causal role in determining the procoagulant status typical of individuals with insulin resistance. The systematic evaluation of all common robustly established genetic determinants of fatty liver disease allowed also to test formally this hypothesis by a Mendelian randomisation approach. The alternative was that the impact of the *PNPLA3* variant was accounted for by direct effects on coagulation independent of liver damage. Despite limitations related to the sample size and lack of invasive assessment of liver fibrosis, results were mainly consistent with progressive NAFLD being a causal determinant of the procoagulant imbalance of individuals with dysmetabolism. However, given these results will require further confirmation in independent and larger cohorts.

*Vice versa*, when we looked at the impact of predisposition to a procoagulant phenotype and the severity of fibrosis, we did not obtain consistent results, possibly because of the relatively low proportion of participants with clinically significant liver fibrosis in this cohort of individuals mostly affected by early-stage NAFLD. However, we observed evidence of a causal association of hypercoagulability with the levels of Pro-C3, the most reliable marker of fibrosis deposition in patients with NAFLD,^35^^,^^36^ from several approaches: (a) directionally consistent and proportional impact of *ABO* variation on vWF, coagulation balance and Pro-C3 levels; (b) evidence that the impact of *ABO* on Pro-C3 was fully mediated by the induction of higher circulating vWF, which was itself strongly associated with Pro-C3; (c) Mendelian randomisation analysis showing that predisposition to higher F8/PC ratio was causally associated with Pro-C3; (d) carriage of the procoagulant variant FVL, acting downstream of F8/PC, being also independently associated with higher Pro-C3. In other words, the Mendelian randomisation framework allowed us to test the directions of the epidemiological association between the procoagulant imbalance and hepatic fibrogenesis, and the results were consistent with a causal role of liver damage in determining a procoagulant phenotype, which in turn would beget fibrogenesis igniting a vicious cycle leading to advanced liver disease. A graphical representation is shown in Fig. S3. These results are potentially important because provide a first link between vWF levels and activation of fibrogenesis, suggesting that higher vWF causes activation of fibrogenesis. Additional studies are required to test whether these findings can be replicated in independent cohorts, and the detailed molecular mechanisms remains to be clarified in experimental studies. However, results are in line with previous evidence indicating that combined carriage of non-O blood group and of FVL were associated with non-invasively assessed fibrosis in a European population.^37^ These results may have translational relevance, as they highlight the possibility that correction of the procoagulant imbalance even at early stage of liver damage may prevent fatty liver progression.^38^^,^^39^ It should also be noted that although in patients with NAFLD collagen deposition correlates with fibrosis stage, hepatic fibrogenesis is a very dynamic process and factors affecting collagen degradation should also be considered,^40^ so that heighted fibrogenesis not always translates into more severe fibrosis, especially at early stage. However, Pro-C3 circulating levels may also reflect collagen deposition in organs other than the liver. Other study limitations include the lack of functional evaluation with thrombin generation procedures, comprehensive evaluation of vWF isoforms and other coagulation factors, and lack of the prospective evaluation of the impact on hard clinical outcomes. In addition, we had a low representation of individuals with progressive NAFLD and in particular of those with severe liver fibrosis and of non-Europeans. However, because of the systematic assessment of individuals with metabolic dysfunction, we could assess the independent impact of metabolic features and liver damage on the coagulation balance, irrespective of the progression to advanced cirrhosis and decompensated liver disease.

In conclusion, the present results suggest that hepatic fat accumulation *per se* does not predispose to hypercoagulability (*e.g.* high F8/PC ratio), whereas fibrosing NAFLD is associated with increased vWF and F8 (as detected by FIB-4), and reduced PC levels (FIB-4 and LSM), resulting in a procoagulant imbalance. Genetic data evaluated in a Mendelian randomisation framework suggest that in individuals with metabolic risk factors, fibrosing NAFLD may play a causal role in the alteration of F8/PC levels, well before the development of advanced liver fibrosis. However, genetic predisposition to hypercoagulability was associated with increased Pro-C3 levels, reflecting hepatic fibrogenesis. Additional studies evaluating larger cohorts, with functional assessment and direct evaluation of liver fibrosis are warranted to further clarify the complex bidirectional interplay between fibrosing NAFLD and the haemostatic balance.

## Financial support

Italian Ministry of Health (Ministero della Salute), Ricerca Finalizzata RF-2016-02364358 (‘Impact of whole exome sequencing on the clinical management of patients with advanced nonalcoholic fatty liver and cryptogenic liver disease’) (LV); Italian Ministry of Health (Ministero della Salute), Rete Cardiologica ‘CV-PREVITAL’ (DP, LV); Italian Ministry of Health, Fondazione IRCCS Ca’ Granda Ospedale Maggiore Policlinico, Ricerca corrente (LV, DP, FP); Fondazione IRCCS Ca’ Granda core COVID-19 Biobank (RC100017A), ‘Liver BIBLE’ (PR-0391) (LV); Innovative Medicines Initiative 2 joint undertaking of European Union’s Horizon 2020 research and innovation programme and EFPIA European Union (EU) Programme Horizon 2020 (under grant agreement No. 777377) for the project LITMUS (LV); The European Union, programme ‘Photonics’ under grant agreement ‘101016726’ (LV); Gilead_IN-IT-989-5790 (LV).

## Authors’ contributions

Conceptualisation: LV, AT, SP, VLM, FP. Methodology: LV, AT, FM. Investigation: VLM, SP, CB, ES, SM, FM, LR, MGC, MF, RDA. Funding acquisition: LV, DP, FP. Project administration: RC. Supervision: LV, AT, VLM, FP, DP. Writing – original draft: LV. Writing – review and editing: LV, AT, FP.

## Data availability statement

The ethical approval of the study does not allow to publicly share individual patients’ genetic data. All data, code, and materials used in the analysis are available upon reasonable request for collaborative studies regulated by materials/data transfer agreements (MTA/DTAs) to the corresponding author.

## Conflicts of interest

The authors declare that they have no conflict of interest relevant to the present study. LV has received speaking fees from MSD, Gilead, AlfaSigma, and AbbVie; served as a consultant for Gilead, Pfizer, AstraZeneca, Novo Nordisk, Intercept, Diatech Pharmacogenetics, and Ionis Pharmaceuticals; and received research grants from Gilead. VLM had received a research grant from Gilead; speaking fees from: Gore, Alfa-Sigma, and CSL-Behring; travel grants from: Takeda and Sanofi.

Please refer to the accompanying ICMJE disclosure forms for further details.
