# Supplementary material for: Clinical and genetic determinants of the fatty liver–coagulation balance interplay in individuals with metabolic dysfunction
Source: JHEP Rep. 2022 Sep 25;4(12):100598. doi: 10.1016/j.jhepr.2022.100598 (PMC9597122; doi:10.1016/j.jhepr.2022.100598)
Supplement: Multimedia component 1 [file mmc1.pdf]

Interplay between coagulation and determinants of liver disease in patients with metabolic dysfunction

Luca Valenti, Armando Tripodi, Vincenzo La Mura, Serena Pelusi, Cristiana Bianco, Erica Scalambrino, Sara Margarita, Francesco Malvestiti, Luisa Ronzoni, Marigrazia Clerici, Roberta D'Ambrosio, Mirella Fraquelli, Rossana Carpani, Daniele Prati, Flora Peyvandi

Table of contents

Supplementary materials and methods..2

Supplementary results...4

Supplementary figures...5

Supplementary tables..7

Supplementary references...12

Supplementary materials and methods

Evaluation of coagulation factors

Upon informed consent blood was collected into vacuum tubes (Vacutainer, Becton Dickinson, Plymouth, UK) containing 1/10 volumes of trisodium citrate 0.109 M; plasma obtained after centrifugation for 20 min at 3,000g was harvested, aliquoted, snap frozen by immersion in liquid nitrogen and stored at −70° until testing.

VWF antigen (VWF:Ag) was measured by a commercial kit (Werfen, Orangeburg, NY) F8 activity was measured with one-stage clotting assays based on aPTT-based assay and F8-deficient plasma. PC levels were measured by specific chromogenic assays (Werfen). Results for the above parameters were expressed as U/dL with reference to a pooled normal plasma arbitrarily assigned the potency of 100 U/dL [1]. D-dimer was measured by latex-based assays (Werfen).

The main outcome we used to assess the regulation of coagulation balance was the F8/PC ratio.

Genotyping and imputation

DNA was extracted from peripheral blood collected at the time of enrolment by the QIASymphony (Qiagen, Milan, Italy). Genotyping was performed by Illumina GlobalScreeningArray (GSA)-24 v3.0 plus Multidisease Array (Illumina, San Diego, CA), which contains 712,189 variants before quality control, at the FIMM Institute for Molecular Medicine (Helsinki, Finland). To maximize genetic coverage, we performed single-nucleotide polymorphism (SNP) imputation on genome build GRCh38 using the Michigan Imputation Server and 194,512 haplotypes generated by the Trans-Omics for Precision Medicine (TOPMed) program (freeze 5) [2]. At the time of analysis, genomic data passing quality control were available for 581 patients.

Mendelian randomization

To assess the reciprocal causal relationship between fatty liver disease and alteration in the coagulation balance, we exploited Mendelian randomization [3, 4]. Mendelian randomization is a framework exploiting human genetic variation to understand if a trait is a causally related risk factor for another trait of interest and is considered the most appropriate tool to assess causality when randomized controlled trials are not feasible. This analysis is based on the idea that because the assignment of alleles is random at conception independently of confounders, genetic variation influencing a trait can be used to assess causality against another trait of interest. Briefly, to estimate the causal relationship between genetic predisposition to NAFLD and the coagulation balance, we used the most established risk variants for NAFLD as instruments in a Mendelian randomization analysis. To estimate the impact of genetic predisposition to NAFLD in risk models, we used the polygenic risk score of hepatic fat content (PRS-HFC), a robust genetic instrument calculated by summing the number of the NAFLD-predisposing alleles in *PNPLA3-TM6SF2-MBOAT7-GCKR* weighted by their effect size on hepatic fat content [5], quantified by the reference standard in the general population [6], further adjusted for variation in *HSD17B13* (PRS-5) [3]. For Mendelian randomization, we included novel loci associated with NAFLD at *TRIB1, APOE*, *GPAM* and *ATG7* [7, 8]. To estimate the causal impact of genetic predisposition to hypercoagulability, we considered the loci associated with F8 at genomewide level in a recent trans-ethnic meta-analysis [9], which were not monomorphic and passed quality control in our cohort. Based on

these, we also calculated a polygenic risk score of F8 levels (PRS-F8) for inclusion in risk models. For the downstream outcome of liver fibrosis, we also considered the rs6025 (Factor V Leiden) and rs1799963 (Prothrombin 20210 variant) SNPs. The causal association of liver damage with coagulation balance and *vice versa* was estimated by robust Mendelian randomization approaches, using coefficients of association of genetic risk variant with the explanatory variables and outcomes determined in the study cohort (adjusted, for age, sex, BMI , and ancestry), taking into account the possible pleiotropic effect of genetic risk variants by the MendelianRandomization R package [10].

Supplementary results

Finally, we exploited a Mendelian randomization approach to investigate the causality and direction of the epidemiological association between liver fibrosis and the procoagulant status. Since the main fatty liver determinant *PNPLA3* p.I148M was associated with F8/PC ratio, we started by assessing the causal association between liver disease predisposition and the coagulation balance. Results are presented in Table S3 and in Figure S1. For this analysis, we focused on classic Inverse variance weighted (IVW) methods, because they gave consistent estimates of causality across the different scores of liver fibrosis. When using robust and penalized corrections to adjust for possible pleiotropy and the uncertainties of estimates of the effect of single variants, IVW-based approaches were all consistent with the existence of a causal association between genetic predisposition to progressive fatty liver disease and procoagulant imbalance ($p<0.05$, Table S3 and Figure S1A). Other estimates (e.g. Median-based or Egger's) were directionally consistent, but not statistically significant (shown e.g. for FNI in Figure S1B).

Next, we looked at the impact of genetic predisposition to increased F8/PC ratio on liver fibrosis (Table S4). We did not observe a consistent pattern of association of F8/PC with fibrosis. No association was detected for FIB-4, a tendency for a positive association for LSM, and for protective association for the FNI score, limited to IVW-based methods. By looking at Pro-C3 measurement, we observed a causal association of genetic predisposition to procoagulant imbalance with this fibrogenesis biomarker at most estimates (Table S4, right column and Figure S2). Robust estimates were directionally consistent (Figure S2B), with no evidence that they were skewed by bias related to unbalanced heterogeneity (MR-Egger intercept, $p>0.05$). However, after excluding the *ABO* locus outlier from the analyses the causal association of F8/PC with FNI and Pro-C3 was lost ($p>0.2$ for alle approaches), leaving open the possibility that it might be driven by pleiotropic effects of ABO.

Supplementary figures

Fig. S1. Causal association between risk factors for progressive NAFLD and alteration of the coagulation balance in the Liver-Bible-2021 cohort (n=591). In this case, the FNI index of fibrosing NAFLD was set as the explanatory variable, whereas the F8/PC ratio as the outcome. A) Impact of single NAFLD risk variants on fibrosis and the coagulation balance; estimates and SE are shown; B) Estimates of the causal association of predisposition to progressive NAFLD and procoagulant imbalance by different modern Mendelian randomization methods.

Figure S1

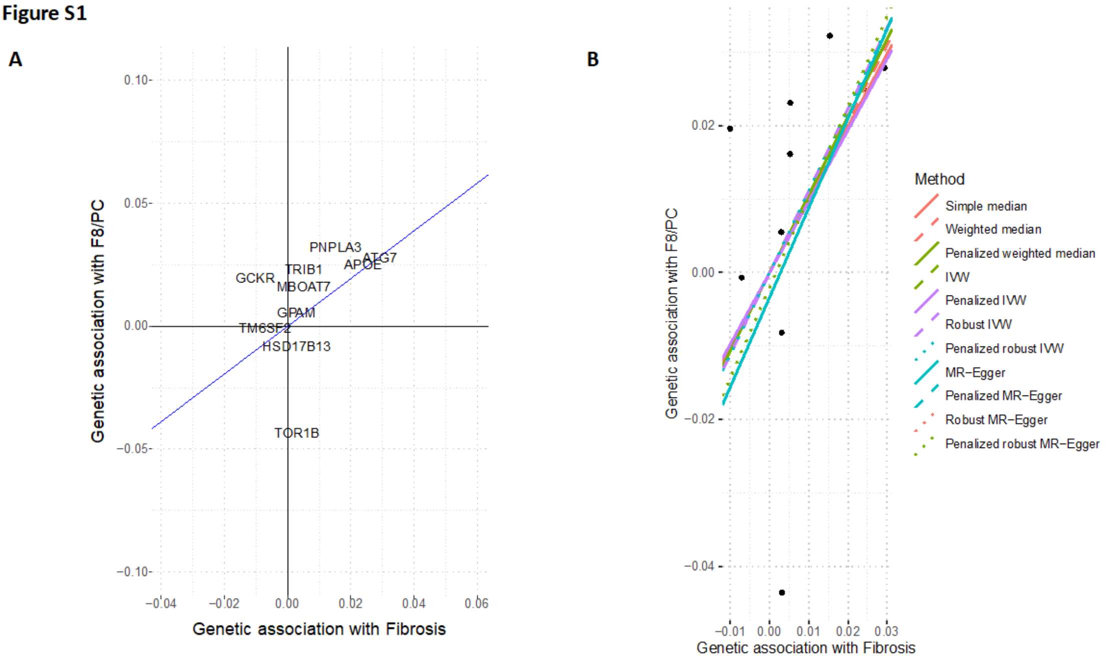

Fig. S2. Causal association between genetic determinants of the F8/PC ratio and Pro-C3 circulating levels in the Liver-Bible-2021 cohort (n=591). IA) Impact of single coagulation variants on the F8/PC ratio and Pro-C3; estimates and SE are shown; B) Estimates of the causal association of predisposition to procoagulant imbalance and heightened fibrogenesis by different modern Mendelian randomization methods.

Figure S2

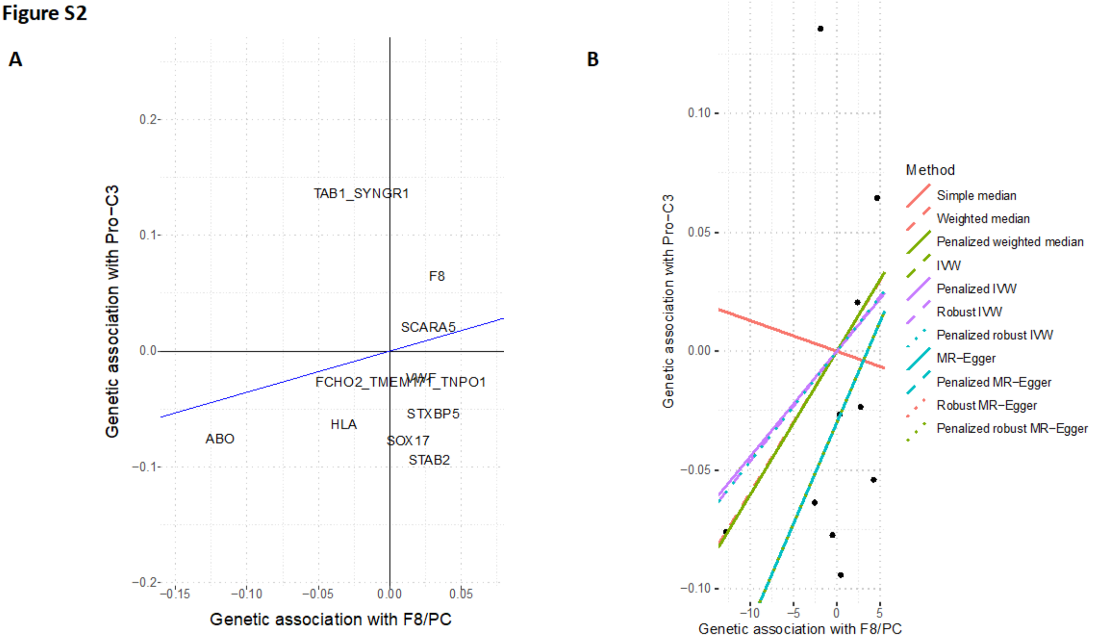

Fig. S3. Graphical overview of the relationship between metabolic alterations, coagulation balance and liver disease and role of genetic factors based on epidemiological and Mendelian randomization analysis conducted in the present study.

Graphical abstract (Figure S3)

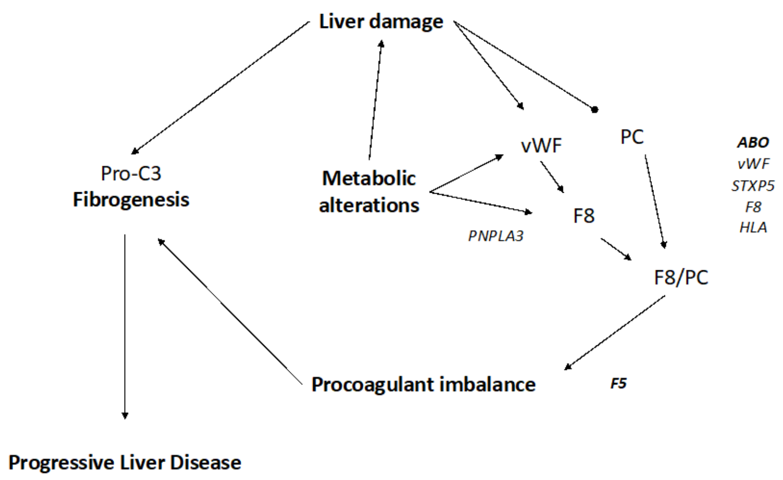

Supplementary tables

Table S1. Frequency distribution and methodology of determination of the genetic variants considered in the study.

	median	1st Q	3rd Q								
**PRS-HFC, score**	0,266	0,128	0,457								
**PRS-5, score**	0,223	0,09	0,394								
**PRS-F8, score**	-0,145	-0,414	-0,092								
	**wt/wt**	**%**	**wt/mut**	**%**	**mut/mut**	**%**	**Chr**	**VAF**	**HWE**	**Determination**	**R2**
***PNPLA3* p.I148M, alleles**	310	53,2	224	38,7	47	8,1	22	0,27	0,47	typed	
***TM6SF2*, p.E167K alleles**	535	0,92	45	0,08	1	0,002	19	0,04	0,96	typed	
***MBOAT7* rs641737, T alleles**	162	27,9	296	51	123	21,1	19	0,5	0,24	typed	
**GCKR, p.P446L, T alleles**	126	21,6	272	46,8	183	31,6	2	0,55	0,19	typed	
**HSD17B17 rs87310240, TA alleles**	343	59,1	208	35,8	30	5,1	4	0,23	0,83	imputed	0,984
**rs7135039 *vWF*, T alleles**	282	48,6	236	40,6	63	10,8	12	0,31	0,2	imputed	0,936
**rs4981022 *STAB2*, A alleles**	49	8,4	232	40	300	51,6	12	0,72	0,66	imputed	0,94
**rs137631 *TAB1-SYNGR1*, C alleles**	8	1,4	122	21	451	77,6	22	0,88	0,94	imputed	0,777
**rs548630 *FCHO2-TMEM171-TNPO1*, C alleles**	109	18,7	280	48,3	192	33	5	0,57	0,7	imputed	0,865
**rs9271597 *HLA,* A alleles**	270	46,4	244	42,1	67	11,5	6	0,33	0,3	imputed	0,999
**rs9399599 *STXBP5,* T alleles**	133	23	298	51,2	150	25,8	6	0,51	0,52	typed	
**rs7816579 *SCARA5,* G alleles**	39	0,7	204	35	338	58,3	8	0,76	0,28	imputed	0,866
**rs10102164 *SOX17-RP1,* A alleles**	429	73,9	138	23,7	14	2,4	8	0,14	0,47	typed	
**rs687289 *ABO,* G alleles**	76	0,13	257	44,2	248	42,8	9	0,65	0,46	typed	
**rs150926226 *TMLHE-F8,* C alleles**	61	10,5	12	0,2	508	87,4	X	0,88	NA	imputed	0,673
**rs6025 *FVLeiden*, alleles**	561	96,6	20	3,4	0	0	1	0,02	0,67	typed	
**rs1799963 *PT20210*, alleles**	559	96,2	21	3,2	1	0,002	11	0,02	0,1	typed	

Table S2. Independent determinants of fibrosis, as estimated by the FIB-4 score, liver stiffness measurement (LSM, kPa), and Fibrosing NASH Index (FNI) in the 581 individuals of the LIVER-BIBLE-2021 cohort with coagulation and genetic data available.

	**FIB-4, score**						**LSM, kPa**						**FNI, score**					
	**Model 1**			**Model 2**			**Model 1**			**Model 2**			**Model 1**			**Model 2**		
	**Estimate**	**SE**	**P-value**	**Estimate**	**SE**	**P-value**	**Estimate**	**SE**	**P value**	**Estimate**	**SE**	**P-value**	**Estimate**	**SE**	**P-value**	**Estimate**	**SE**	**P-value**
Age, years	0,02	0,00	**9,00E-23**	0,02	0,00	**4,00E-22**	0,004	0,008	0,61	0,004	0,008	0,61	-0,001	0,00	**0,022**	-0,002	0,00	**0,01**
SEX, F	-0,04	0,02	**0,0196**	-0,05	0,02	**0,015**	-0,49	0,07	**1,00E-11**	-0,49	0,07	**3,00E-11**	-0,01	0,01	**0,0149**	-0,01	0,01	**0,0093**
BMI, Kg/m2	0,00	0,00	0,33	0,00	0,00	0,3	0,10	0,02	**1,00E-08**	0,10	0,02	**1,00E-08**	0,00	0,00	0,13	0,00	0,00	0,17
Insulin, mU/l	-0,004	0,00	**0,0157**	-0,004	0,00	**0,016**	0,02	0,01	**0,0008**	0,02	0,01	**0,0007**	0,001	0,000	**0,023**	0,001	0,000	**0,024**
F8/PC, ratio	0,17	0,05	**0,0005**	0,21	0,05	**7,00E-05**	0,52	0,18	**0,0046**	0,51	0,20	**0,0092**	0,02	0,01	0,12	0,04	0,02	**0,012**
PRS-5, score				0,06	0,07	0,42				-0,06	0,26	0,81				0,06	0,03	**0,031**
PRS-F8, score				-0,26	0,14	0,066				0,20	0,52	0,71				-0,13	0,04	**0,0015**
rs6025 *FVLeiden*, alleles				-0,06	0,07	0,38				-0,29	0,28	0,29				-0,04	0,02	0,063

Table S3. Causal association of liver damage indices with procoagulant alteration of the hemostatic balance (F8/PC ratio).

	FIB4 on F8/PC ratio					LSM on F8/PC ratio					FNI on F8/PC ratio				
**Method**	**Estimate**	**Std Error**	**95% CI**		**P value**	**Estimate**	**Std Error**	**95% CI**		**P value**	**Estimate**	**Std Error**	**95% CI**		**P value**
IVW	0.308	0.256	-0.194	0.809	0.229	0.173	0.073	0.03	0.315	**0.017**	0.969	0.593	-0.192	2.131	0.102
Penalized IVW	0.308	0.256	-0.194	0.809	0.229	0.173	0.073	0.03	0.315	**0.017**	0.969	0.593	-0.192	2.131	0.102
Robust IVW	0.296	0.119	0.063	0.53	**0.013**	0.168	0.034	0.102	0.234	**0**	1.112	0.568	-0.001	2.224	**0.050**
Penalized robust IVW	0.296	0.119	0.063	0.53	**0.013**	0.168	0.034	0.102	0.234	**0**	1.112	0.568	-0.001	2.224	**0.050**

Table S4. Causal association of procoagulant alteration of the hemostatic balance (F8/PC ratio) with fibrosis indices and fibrogenesis (Pro-C3).

	F8/PC on FIB-4					F8/PC on LSM					F8/PC on FNI					vWF/F8 on Pro-C3				
**Method**	**Estimate**	**Std Error**	**95% CI**		**P-value**	**Estimate**	**Std Error**	**95% CI**		**P-value**	**Estimate**	**Std Error**	**95% CI**		**P-value**	**Estimate**	**Std Error**	**95% CI**		**P-value**
Simple median	0.001	0.004	-0.007	0.01	0.779	0.017	0.014	-0.011	0.044	0.229	0	0.001	-0.002	0.002	0.944	-0.001	0.01	-0.02	0.018	0.893
Weighted median	-0.001	0.002	-0.004	0.002	0.424	0.008	0.006	-0.003	0.019	0.15	-0.001	0	-0.002	0	0.139	0.006	0.003	0	0.012	0.054
Penalized weighted median	-0.001	0.002	-0.004	0.002	0.425	0.008	0.006	-0.003	0.019	0.15	-0.001	0	-0.002	0	0.139	0.006	0.003	0	0.012	**0.05**
IVW	-0.001	0.002	-0.004	0.003	0.666	0.01	0.005	0	0.02	**0.043**	-0.001	0	-0.002	0	**0.035**	0.004	0.004	-0.003	0.012	0.274
Penalized IVW	-0.001	0.002	-0.004	0.003	0.666	0.01	0.005	0	0.02	**0.043**	-0.001	0	-0.002	0	**0.035**	0.004	0.004	-0.003	0.012	0.274
Robust IVW	-0.001	0.001	-0.003	0.001	0.423	0.01	0.002	0.005	0.015	**0**	-0.001	0	-0.001	0	**0**	0.005	0.002	0	0.009	**0.032**
Penalized robust IVW	-0.001	0.001	-0.003	0.001	0.423	0.01	0.002	0.005	0.015	**0**	-0.001	0	-0.001	0	**0**	0.005	0.002	0	0.009	**0.032**
MR-Egger	-0.001	0.003	-0.007	0.004	0.612	0.007	0.007	-0.007	0.02	0.348	-0.001	0.001	-0.002	0	0.233	0.008	0.006	-0.002	0.019	0.127
(intercept)	0.004	0.014	-0.022	0.031	0.76	0.027	0.036	-0.043	0.097	0.449	-0.001	0.003	-0.007	0.004	0.634	-0.03	0.028	-0.086	0.026	0.29
Penalized MR-Egger	-0.001	0.003	-0.007	0.004	0.612	0.007	0.007	-0.007	0.02	0.348	-0.001	0.001	-0.002	0	0.233	0.008	0.006	-0.002	0.019	0.127
(intercept)	0.004	0.014	-0.022	0.031	0.76	0.027	0.036	-0.043	0.097	0.449	-0.001	0.003	-0.007	0.004	0.634	-0.03	0.028	-0.086	0.026	0.29
Robust MR-Egger	-0.002	0.001	-0.004	0.001	0.24	0.005	0.006	-0.006	0.016	0.382	-0.001	0.001	-0.003	0.001	0.409	0.008	0.002	0.004	0.013	**0.001**
(intercept)	0.006	0.012	-0.018	0.03	0.625	0.041	0.057	-0.071	0.153	0.473	0.001	0.014	-0.026	0.028	0.949	-0.03	0.025	-0.078	0.018	0.225
Penalized robust MR-Egger	-0.002	0.001	-0.004	0.001	0.24	0.005	0.006	-0.006	0.016	0.382	-0.001	0.001	-0.003	0.001	0.409	0.008	0.002	0.004	0.013	**0.001**
(intercept)	0.006	0.012	-0.018	0.03	0.625	0.041	0.057	-0.071	0.153	0.473	0.001	0.014	-0.026	0.028	0.949	-0.03	0.025	-0.078	0.018	0.225

Supplementary references

[1] Tripodi A, Rossi SC, Clerici M, Merati G, Scalambrino E, Mancini I, et al. Pro-coagulant imbalance in patients with community acquired pneumonia assessed on admission and one month after hospital discharge. Clin Chem Lab Med 2021;59:1699-1708.
[2] Severe Covid GG, Ellinghaus D, Degenhardt F, Bujanda L, Buti M, Albillos A, et al. Genomewide Association Study of Severe Covid-19 with Respiratory Failure. N Engl J Med 2020;383:1522-1534.
[3] Bianco C, Jamialahmadi O, Pelusi S, Baselli G, Dongiovanni P, Zanoni I, et al. Non-invasive stratification of hepatocellular carcinoma risk in non-alcoholic fatty liver using polygenic risk scores. J Hepatol 2021;74:775-782.
[4] Davey Smith G, Hemani G. Mendelian randomization: genetic anchors for causal inference in epidemiological studies. Hum Mol Genet 2014;23:R89-98.
[5] Trepo E, Valenti L. Update on NAFLD genetics: From new variants to the clinic. J Hepatol 2020;72:1196-1209.
[6] Dongiovanni P, Stender S, Pietrelli A, Mancina RM, Cespiati A, Petta S, et al. Causal relationship of hepatic fat with liver damage and insulin resistance in nonalcoholic fatty liver. J Intern Med 2018;283:356-370.
[7] Baselli GA, Jamialahmadi O, Pelusi S, Ciociola E, Malvestiti F, Saracino M, et al. Rare ATG7 genetic variants predispose patients to severe fatty liver disease. J Hepatol 2022.
[8] Jamialahmadi O, Mancina RM, Ciociola E, Tavaglione F, Luukkonen PK, Baselli G, et al. Exome-Wide Association Study on Alanine Aminotransferase Identifies Sequence Variants in the GPAM and APOE Associated With Fatty Liver Disease. Gastroenterology 2021;160:1634-1646 e1637.
[9] Sabater-Lleal M, Huffman JE, de Vries PS, Marten J, Mastrangelo MA, Song C, et al. Genome-Wide Association Transethnic Meta-Analyses Identifies Novel Associations Regulating Coagulation Factor VIII and von Willebrand Factor Plasma Levels. Circulation 2019;139:620-635.
[10] Davies NM, Holmes MV, Davey Smith G. Reading Mendelian randomisation studies: a guide, glossary, and checklist for clinicians. BMJ 2018;362:k601.
