# Supplementary material for: Clinical and genetic determinants of the fatty liver–coagulation balance interplay in individuals with metabolic dysfunction
Source: JHEP Rep. 2022 Sep 25;4(12):100598. doi: 10.1016/j.jhepr.2022.100598 (PMC9597122; doi:10.1016/j.jhepr.2022.100598)
Supplement: Multimedia component 2 [file mmc2.docx]

**Journal of Hepatology**

**CTAT methods**

Tables for a “Complete, Transparent, Accurate and Timely account” (CTAT) are now mandatory for all revised submissions. The aim is to enhance the reproducibility of methods.

- Only include the parts relevant to your study
- Refer to the CTAT in the main text as ‘Supplementary CTAT Table’
- Do not add subheadings
- Add as many rows as needed to include all information
- Only include one item per row

**If the CTAT form is not relevant to your study, please outline the reasons why:**

| NA |
| --- |

- 1. **Antibodies: NA**

| **Name** | **Citation** | **Supplier** | **Cat no.** | **Clone no.** |
| --- | --- | --- | --- | --- |
|  |  |  |  |  |

- 1. **Cell lines: NA**

| **Name** | **Citation** | **Supplier** | **Cat no.** | **Passage no.** | **Authentication test method** |
| --- | --- | --- | --- | --- | --- |
|  |  |  |  |  |  |

- 1. **Organisms: NA**

| **Name** | **Citation** | **Supplier** | **Strain** | **Sex** | **Age** | **Overall n number** |
| --- | --- | --- | --- | --- | --- | --- |
|  |  |  |  |  |  |  |

- 1. **Sequence based reagents: NA**

| **Name** | **Sequence** | **Supplier** |
| --- | --- | --- |
|  |  |  |

- 1. **Biological samples**

| **Description** | **Source** | **Identifier** |
| --- | --- | --- |
| Whole Blood | Fondazione IRCCS Ca’ Granda Ospedale Maggiore Policlinico | Enrolled from June 2019 to June 2021 |

- 1. **Deposited data**

| **Name of repository** | **Identifier** | **Link** |
| --- | --- | --- |
| Mendeley data | DOI: 10.17632/gff2ftfhmr.1 (Reserved) | https://data.mendeley.com/ |

- 1. **Software**

| **Software name** | **Manufacturer** | **Version** |
| --- | --- | --- |
| R | CRAN | 4.0.3 |
| JMP | SAS | 16.0 Pro |

- 1. **Other (e.g. drugs, proteins, vectors etc.) : NA**

|  |  |  |
| --- | --- | --- |
|  |  |  |

- 1. **Please provide the details of the corresponding methods author for the manuscript:**

| **Luca Valenti, MD**  **Precision Medicine – Department of Transfusion Medicine and Hematology**  **Fondazione IRCCS Ca’ Granda Ospedale Maggiore Policlinico**  **via Francesco Sforza 35**  **20122, Milan, Italy**  **Email: luca.valenti@unimi.it** |
| --- |

**2.0 Please confirm for randomised controlled trials all versions of the clinical protocol are included in the submission. These will be published online as supplementary information.**

| **NA** |
| --- |
