# Supplementary material for: Clinical and genetic determinants of the fatty liver–coagulation balance interplay in individuals with metabolic dysfunction
Source: JHEP Rep. 2022 Sep 25;4(12):100598. doi: 10.1016/j.jhepr.2022.100598 (PMC9597122; doi:10.1016/j.jhepr.2022.100598)
Supplement: Multimedia component 3 [file mmc3.pdf]

ICMJE DISCLOSURE FORM

Date: 31 August 2022
Your Name: Luca Vittorio Valenti
Manuscript Title: CLINICAL AND GENETIC DETERMINANTS OF THE FATTY LIVER - COAGULATION BALANCE INTERPLAY IN INDIVIDUALS WITH METABOLIC DYSFUNCTION

Manuscript number (if known): HEPR-D-22-00290-R1

In the interest of transparency, we ask you to disclose all relationships/activities/interests listed below that are related to the content of your manuscript. "Related" means any relation with for-profit or not-for-profit third parties whose interests may be affected by the content of the manuscript. Disclosure represents a commitment to transparency and does not necessarily indicate a bias. If you are in doubt about whether to list a relationship/activity/interest, it is preferable that you do so.

The following questions apply to the author's relationships/activities/interests as they relate to the current manuscript only.

The author's relationships/activities/interests should be defined broadly. For example, if your manuscript pertains to the epidemiology of hypertension, you should declare all relationships with manufacturers of antihypertensive medication, even if that medication is not mentioned in the manuscript.

In item #1 below, report all support for the work reported in this manuscript without time limit. For all other items, the time frame for disclosure is the past 36 months.

		**Name all entities with whom you have this relationship or indicate none (add rows as needed)**	**Specifications/Comments (e.g., if payments were made to you or to your institution)**
		**Time frame: Since the initial planning of the work**	
1	All support for the present manuscript (e.g., funding, provision of study materials, medical writing, article processing charges, etc.) **No time limit for this item.**	**X** None	
		**Time frame: past 36 months**	
2	Grants or contracts from any entity (if not indicated in item #1 above).	____None	Gilead Sciences
3	Royalties or licenses	**X** None	

4	Consulting fees	____None	Gilead, Pfizer, Astra Zeneca, Novo Nordisk, Intercept pharmaceuticals, Diatech Pharmacogenetics, IONIS, Boehringer Ingelheim
5	Payment or honoraria for lectures, presentations, speakers bureaus, manuscript writing or educational events	____None	MSD, Gilead, AlfaSigma, AbbVie, Viatris
6	Payment for expert testimony	**X** None	
7	Support for attending meetings and/or travel	____None	Gilead Science
8	Patents planned, issued or pending	**X** None	
9	Participation on a Data Safety Monitoring Board or Advisory Board	____None	Intercept, Pfizer, Gilead, Novo Nordisk
10	Leadership or fiduciary role in other board, society, committee or advocacy group, paid or unpaid	**X** None	
11	Stock or stock options	**X** None	
12	Receipt of equipment, materials, drugs, medical writing, gifts or other services	**X** None	
13	Other financial or non-financial interests	**X** None	

Please place an "X" next to the following statement to indicate your agreement:

X I certify that I have answered every question and have not altered the wording of any of the questions on this form.

ICMJE DISCLOSURE FORM

Date: 31 August 2022
Your Name: Serena Pelusi
Manuscript Title: CLINICAL AND GENETIC DETERMINANTS OF THE FATTY LIVER - COAGULATION BALANCE INTERPLAY IN INDIVIDUALS WITH METABOLIC DYSFUNCTION

Manuscript number (if known): HEPR-D-22-00290-R1

In the interest of transparency, we ask you to disclose all relationships/activities/interests listed below that are related to the content of your manuscript. "Related" means any relation with for-profit or not-for-profit third parties whose interests may be affected by the content of the manuscript. Disclosure represents a commitment to transparency and does not necessarily indicate a bias. If you are in doubt about whether to list a relationship/activity/interest, it is preferable that you do so.

The following questions apply to the author's relationships/activities/interests as they relate to the current manuscript only.

The author's relationships/activities/interests should be defined broadly. For example, if your manuscript pertains to the epidemiology of hypertension, you should declare all relationships with manufacturers of antihypertensive medication, even if that medication is not mentioned in the manuscript.

In item #1 below, report all support for the work reported in this manuscript without time limit. For all other items, the time frame for disclosure is the past 36 months.

		**Name all entities with whom you have this relationship or indicate none (add rows as needed)**	**Specifications/Comments (e.g., if payments were made to you or to your institution)**
		**Time frame: Since the initial planning of the work**	
1	All support for the present manuscript (e.g., funding, provision of study materials, medical writing, article processing charges, etc.) **No time limit for this item.**	**X** None	
		**Time frame: past 36 months**	
2	Grants or contracts from any entity (if not indicated in item #1 above).	____None	
3	Royalties or licenses	- None	

4	Consulting fees	____None	
5	Payment or honoraria for lectures, presentations, speakers bureaus, manuscript writing or educational events	____None	
6	Payment for expert testimony	**X** None	
7	Support for attending meetings and/or travel	____None	
8	Patents planned, issued or pending	**X** None	
9	Participation on a Data Safety Monitoring Board or Advisory Board	____None	
10	Leadership or fiduciary role in other board, society, committee or advocacy group, paid or unpaid	**X** None	
11	Stock or stock options	**X** None	
12	Receipt of equipment, materials, drugs, medical writing, gifts or other services	**X** None	
13	Other financial or non-financial interests	**X** None	

Please place an "X" next to the following statement to indicate your agreement:

X I certify that I have answered every question and have not altered the wording of any of the questions on this form.

ICMJE DISCLOSURE FORM

Date: 31 August 2022
Your Name: Cristiana Bianco
Manuscript Title: CLINICAL AND GENETIC DETERMINANTS OF THE FATTY LIVER - COAGULATION BALANCE INTERPLAY IN INDIVIDUALS WITH METABOLIC DYSFUNCTION

Manuscript number (if known): HEPR-D-22-00290-R1

In the interest of transparency, we ask you to disclose all relationships/activities/interests listed below that are related to the content of your manuscript. "Related" means any relation with for-profit or not-for-profit third parties whose interests may be affected by the content of the manuscript. Disclosure represents a commitment to transparency and does not necessarily indicate a bias. If you are in doubt about whether to list a relationship/activity/interest, it is preferable that you do so.

The following questions apply to the author's relationships/activities/interests as they relate to the current manuscript only.

The author's relationships/activities/interests should be defined broadly. For example, if your manuscript pertains to the epidemiology of hypertension, you should declare all relationships with manufacturers of antihypertensive medication, even if that medication is not mentioned in the manuscript.

In item #1 below, report all support for the work reported in this manuscript without time limit. For all other items, the time frame for disclosure is the past 36 months.

		**Name all entities with whom you have this relationship or indicate none (add rows as needed)**	**Specifications/Comments (e.g., if payments were made to you or to your institution)**
		**Time frame: Since the initial planning of the work**	
1	All support for the present manuscript (e.g., funding, provision of study materials, medical writing, article processing charges, etc.) **No time limit for this item.**	**X** None	
		**Time frame: past 36 months**	
2	Grants or contracts from any entity (if not indicated in item #1 above).	____None	
3	Royalties or licenses	- None	

4	Consulting fees	____None	
5	Payment or honoraria for lectures, presentations, speakers bureaus, manuscript writing or educational events	____None	
6	Payment for expert testimony	**X** None	
7	Support for attending meetings and/or travel	____None	
8	Patents planned, issued or pending	**X** None	
9	Participation on a Data Safety Monitoring Board or Advisory Board	____None	
10	Leadership or fiduciary role in other board, society, committee or advocacy group, paid or unpaid	**X** None	
11	Stock or stock options	**X** None	
12	Receipt of equipment, materials, drugs, medical writing, gifts or other services	**X** None	
13	Other financial or non-financial interests	**X** None	

Please place an "X" next to the following statement to indicate your agreement:

X I certify that I have answered every question and have not altered the wording of any of the questions on this form.

ICMJE DISCLOSURE FORM

Date: 31 August 2022
Your Name: Marigrazia Clerici
Manuscript Title: CLINICAL AND GENETIC DETERMINANTS OF THE FATTY LIVER - COAGULATION BALANCE INTERPLAY IN INDIVIDUALS WITH METABOLIC DYSFUNCTION

Manuscript number (if known): HEPR-D-22-00290-R1

In the interest of transparency, we ask you to disclose all relationships/activities/interests listed below that are related to the content of your manuscript. "Related" means any relation with for-profit or not-for-profit third parties whose interests may be affected by the content of the manuscript. Disclosure represents a commitment to transparency and does not necessarily indicate a bias. If you are in doubt about whether to list a relationship/activity/interest, it is preferable that you do so.

The following questions apply to the author's relationships/activities/interests as they relate to the current manuscript only.

The author's relationships/activities/interests should be defined broadly. For example, if your manuscript pertains to the epidemiology of hypertension, you should declare all relationships with manufacturers of antihypertensive medication, even if that medication is not mentioned in the manuscript.

In item #1 below, report all support for the work reported in this manuscript without time limit. For all other items, the time frame for disclosure is the past 36 months.

		**Name all entities with whom you have this relationship or indicate none (add rows as needed)**	**Specifications/Comments (e.g., if payments were made to you or to your institution)**
**Time frame: Since the initial planning of the work**			
1	All support for the present manuscript (e.g., funding, provision of study materials, medical writing, article processing charges, etc.) **No time limit for this item.**	**X** None	
**Time frame: past 36 months**			
2	Grants or contracts from any entity (if not indicated in item #1 above).	____None	
3	Royalties or licenses	- None	

4	Consulting fees	____None	
5	Payment or honoraria for lectures, presentations, speakers bureaus, manuscript writing or educational events	____None	
6	Payment for expert testimony	**X** None	
7	Support for attending meetings and/or travel	____None	
8	Patents planned, issued or pending	**X** None	
9	Participation on a Data Safety Monitoring Board or Advisory Board	____None	
10	Leadership or fiduciary role in other board, society, committee or advocacy group, paid or unpaid	**X** None	
11	Stock or stock options	**X** None	
12	Receipt of equipment, materials, drugs, medical writing, gifts or other services	**X** None	
13	Other financial or non-financial interests	**X** None	

Please place an "X" next to the following statement to indicate your agreement:

X I certify that I have answered every question and have not altered the wording of any of the questions on this form.

ICMJE DISCLOSURE FORM

Date: 31 August 2022
Your Name: Luisa Ronzoni
Manuscript Title: CLINICAL AND GENETIC DETERMINANTS OF THE FATTY LIVER - COAGULATION BALANCE INTERPLAY IN INDIVIDUALS WITH METABOLIC DYSFUNCTION

Manuscript number (if known): HEPR-D-22-00290-R1

In the interest of transparency, we ask you to disclose all relationships/activities/interests listed below that are related to the content of your manuscript. "Related" means any relation with for-profit or not-for-profit third parties whose interests may be affected by the content of the manuscript. Disclosure represents a commitment to transparency and does not necessarily indicate a bias. If you are in doubt about whether to list a relationship/activity/interest, it is preferable that you do so.

The following questions apply to the author's relationships/activities/interests as they relate to the current manuscript only.

The author's relationships/activities/interests should be defined broadly. For example, if your manuscript pertains to the epidemiology of hypertension, you should declare all relationships with manufacturers of antihypertensive medication, even if that medication is not mentioned in the manuscript.

In item #1 below, report all support for the work reported in this manuscript without time limit. For all other items, the time frame for disclosure is the past 36 months.

		**Name all entities with whom you have this relationship or indicate none (add rows as needed)**	**Specifications/Comments (e.g., if payments were made to you or to your institution)**
		**Time frame: Since the initial planning of the work**	
1	All support for the present manuscript (e.g., funding, provision of study materials, medical writing, article processing charges, etc.) **No time limit for this item.**	**X** None	
		**Time frame: past 36 months**	
2	Grants or contracts from any entity (if not indicated in item #1 above).	____None	
3	Royalties or licenses	**-** None	

4	Consulting fees	____None	
5	Payment or honoraria for lectures, presentations, speakers bureaus, manuscript writing or educational events	____None	
6	Payment for expert testimony	**X** None	
7	Support for attending meetings and/or travel	____None	
8	Patents planned, issued or pending	**X** None	
9	Participation on a Data Safety Monitoring Board or Advisory Board	____None	
10	Leadership or fiduciary role in other board, society, committee or advocacy group, paid or unpaid	**X** None	
11	Stock or stock options	**X** None	
12	Receipt of equipment, materials, drugs, medical writing, gifts or other services	**X** None	
13	Other financial or non-financial interests	**X** None	

Please place an "X" next to the following statement to indicate your agreement:

X I certify that I have answered every question and have not altered the wording of any of the questions on this form.

ICMJE DISCLOSURE FORM

Date: 31 August 2022
Your Name: Rossana Carpani
Manuscript Title: CLINICAL AND GENETIC DETERMINANTS OF THE FATTY LIVER - COAGULATION BALANCE INTERPLAY IN INDIVIDUALS WITH METABOLIC DYSFUNCTION

Manuscript number (if known): HEPR-D-22-00290-R1

In the interest of transparency, we ask you to disclose all relationships/activities/interests listed below that are related to the content of your manuscript. "Related" means any relation with for-profit or not-for-profit third parties whose interests may be affected by the content of the manuscript. Disclosure represents a commitment to transparency and does not necessarily indicate a bias. If you are in doubt about whether to list a relationship/activity/interest, it is preferable that you do so.

The following questions apply to the author's relationships/activities/interests as they relate to the current manuscript only.

The author's relationships/activities/interests should be defined broadly. For example, if your manuscript pertains to the epidemiology of hypertension, you should declare all relationships with manufacturers of antihypertensive medication, even if that medication is not mentioned in the manuscript.

In item #1 below, report all support for the work reported in this manuscript without time limit. For all other items, the time frame for disclosure is the past 36 months.

		Name all entities with whom you have this relationship or indicate none (add rows as needed)	Specifications/Comments (e.g., if payments were made to you or to your institution)
**Time frame: Since the initial planning of the work**			
1	All support for the present manuscript (e.g., funding, provision of study materials, medical writing, article processing charges, etc.) **No time limit for this item.**	**X** None	
**Time frame: past 36 months**			
2	Grants or contracts from any entity (if not indicated in item #1 above).	____None	
3	Royalties or licenses	- None	

4	Consulting fees	____None	
5	Payment or honoraria for lectures, presentations, speakers bureaus, manuscript writing or educational events	____None	
6	Payment for expert testimony	**X** None	
7	Support for attending meetings and/or travel	____None	
8	Patents planned, issued or pending	**X** None	
9	Participation on a Data Safety Monitoring Board or Advisory Board	____None	
10	Leadership or fiduciary role in other board, society, committee or advocacy group, paid or unpaid	**X** None	
11	Stock or stock options	**X** None	
12	Receipt of equipment, materials, drugs, medical writing, gifts or other services	**X** None	
13	Other financial or non-financial interests	**X** None	

Please place an "X" next to the following statement to indicate your agreement:

X I certify that I have answered every question and have not altered the wording of any of the questions on this form.

ICMJE DISCLOSURE FORM

Date: 31 August 2022
Your Name: Francesco Malvestiti
Manuscript Title: CLINICAL AND GENETIC DETERMINANTS OF THE FATTY LIVER - COAGULATION BALANCE INTERPLAY IN INDIVIDUALS WITH METABOLIC DYSFUNCTION

Manuscript number (if known): HEPR-D-22-00290-R1

In the interest of transparency, we ask you to disclose all relationships/activities/interests listed below that are related to the content of your manuscript. "Related" means any relation with for-profit or not-for-profit third parties whose interests may be affected by the content of the manuscript. Disclosure represents a commitment to transparency and does not necessarily indicate a bias. If you are in doubt about whether to list a relationship/activity/interest, it is preferable that you do so.

The following questions apply to the author's relationships/activities/interests as they relate to the current manuscript only.

The author's relationships/activities/interests should be defined broadly. For example, if your manuscript pertains to the epidemiology of hypertension, you should declare all relationships with manufacturers of antihypertensive medication, even if that medication is not mentioned in the manuscript.

In item #1 below, report all support for the work reported in this manuscript without time limit. For all other items, the time frame for disclosure is the past 36 months.

		Name all entities with whom you have this relationship or indicate none (add rows as needed)	Specifications/Comments (e.g., if payments were made to you or to your institution)
**Time frame: Since the initial planning of the work**			
1	All support for the present manuscript (e.g., funding, provision of study materials, medical writing, article processing charges, etc.) **No time limit for this item.**	**X** None	
**Time frame: past 36 months**			
2	Grants or contracts from any entity (if not indicated in item #1 above).	____None	
3	Royalties or licenses	- None	

4	Consulting fees	____None	
5	Payment or honoraria for lectures, presentations, speakers bureaus, manuscript writing or educational events	____None	
6	Payment for expert testimony	**X** None	
7	Support for attending meetings and/or travel	____None	
8	Patents planned, issued or pending	**X** None	
9	Participation on a Data Safety Monitoring Board or Advisory Board	____None	
10	Leadership or fiduciary role in other board, society, committee or advocacy group, paid or unpaid	**X** None	
11	Stock or stock options	**X** None	
12	Receipt of equipment, materials, drugs, medical writing, gifts or other services	**X** None	
13	Other financial or non-financial interests	**X** None	

Please place an "X" next to the following statement to indicate your agreement:

X I certify that I have answered every question and have not altered the wording of any of the questions on this form.

ICMJE DISCLOSURE FORM

Date: 31 August 2022
Your Name: Erica Scalambrino
Manuscript Title: CLINICAL AND GENETIC DETERMINANTS OF THE FATTY LIVER - COAGULATION BALANCE INTERPLAY IN INDIVIDUALS WITH METABOLIC DYSFUNCTION

Manuscript number (if known): HEPR-D-22-00290-R1

In the interest of transparency, we ask you to disclose all relationships/activities/interests listed below that are related to the content of your manuscript. "Related" means any relation with for-profit or not-for-profit third parties whose interests may be affected by the content of the manuscript. Disclosure represents a commitment to transparency and does not necessarily indicate a bias. If you are in doubt about whether to list a relationship/activity/interest, it is preferable that you do so.

The following questions apply to the author's relationships/activities/interests as they relate to the current manuscript only.

The author's relationships/activities/interests should be defined broadly. For example, if your manuscript pertains to the epidemiology of hypertension, you should declare all relationships with manufacturers of antihypertensive medication, even if that medication is not mentioned in the manuscript.

In item #1 below, report all support for the work reported in this manuscript without time limit. For all other items, the time frame for disclosure is the past 36 months.

		**Name all entities with whom you have this relationship or indicate none (add rows as needed)**	**Specifications/Comments (e.g., if payments were made to you or to your institution)**
**Time frame: Since the initial planning of the work**			
1	All support for the present manuscript (e.g., funding, provision of study materials, medical writing, article processing charges, etc.) **No time limit for this item.**	**X** None	
**Time frame: past 36 months**			
2	Grants or contracts from any entity (if not indicated in item #1 above).	____None	
3	Royalties or licenses	- None	

4	Consulting fees	____None	
5	Payment or honoraria for lectures, presentations, speakers bureaus, manuscript writing or educational events	____None	
6	Payment for expert testimony	**X** None	
7	Support for attending meetings and/or travel	____None	
8	Patents planned, issued or pending	**X** None	
9	Participation on a Data Safety Monitoring Board or Advisory Board	____None	
10	Leadership or fiduciary role in other board, society, committee or advocacy group, paid or unpaid	**X** None	
11	Stock or stock options	**X** None	
12	Receipt of equipment, materials, drugs, medical writing, gifts or other services	**X** None	
13	Other financial or non-financial interests	**X** None	

Please place an "X" next to the following statement to indicate your agreement:

X I certify that I have answered every question and have not altered the wording of any of the questions on this form.

ICMJE DISCLOSURE FORM

Date: 31 August 2022
Your Name: Sara Margarita
Manuscript Title: CLINICAL AND GENETIC DETERMINANTS OF THE FATTY LIVER - COAGULATION BALANCE INTERPLAY IN INDIVIDUALS WITH METABOLIC DYSFUNCTION

Manuscript number (if known): HEPR-D-22-00290-R1

In the interest of transparency, we ask you to disclose all relationships/activities/interests listed below that are related to the content of your manuscript. "Related" means any relation with for-profit or not-for-profit third parties whose interests may be affected by the content of the manuscript. Disclosure represents a commitment to transparency and does not necessarily indicate a bias. If you are in doubt about whether to list a relationship/activity/interest, it is preferable that you do so.

The following questions apply to the author's relationships/activities/interests as they relate to the current manuscript only.

The author's relationships/activities/interests should be defined broadly. For example, if your manuscript pertains to the epidemiology of hypertension, you should declare all relationships with manufacturers of antihypertensive medication, even if that medication is not mentioned in the manuscript.

In item #1 below, report all support for the work reported in this manuscript without time limit. For all other items, the time frame for disclosure is the past 36 months.

		Name all entities with whom you have this relationship or indicate none (add rows as needed)	Specifications/Comments (e.g., if payments were made to you or to your institution)
**Time frame: Since the initial planning of the work**			
1	All support for the present manuscript (e.g., funding, provision of study materials, medical writing, article processing charges, etc.) **No time limit for this item.**	**X** None	
**Time frame: past 36 months**			
2	Grants or contracts from any entity (if not indicated in item #1 above).	____None	
3	Royalties or licenses	- None	

4	Consulting fees	____None	
5	Payment or honoraria for lectures, presentations, speakers bureaus, manuscript writing or educational events	____None	
6	Payment for expert testimony	**X** None	
7	Support for attending meetings and/or travel	____None	
8	Patents planned, issued or pending	**X** None	
9	Participation on a Data Safety Monitoring Board or Advisory Board	____None	
10	Leadership or fiduciary role in other board, society, committee or advocacy group, paid or unpaid	**X** None	
11	Stock or stock options	**X** None	
12	Receipt of equipment, materials, drugs, medical writing, gifts or other services	**X** None	
13	Other financial or non-financial interests	**X** None	

Please place an "X" next to the following statement to indicate your agreement:

X I certify that I have answered every question and have not altered the wording of any of the questions on this form.

ICMJE DISCLOSURE FORM

Date: 31 August 2022
Your Name: Roberta D'Ambrosio
Manuscript Title: CLINICAL AND GENETIC DETERMINANTS OF THE FATTY LIVER - COAGULATION BALANCE INTERPLAY IN INDIVIDUALS WITH METABOLIC DYSFUNCTION

Manuscript number (if known): HEPR-D-22-00290-R1

In the interest of transparency, we ask you to disclose all relationships/activities/interests listed below that are related to the content of your manuscript. "Related" means any relation with for-profit or not-for-profit third parties whose interests may be affected by the content of the manuscript. Disclosure represents a commitment to transparency and does not necessarily indicate a bias. If you are in doubt about whether to list a relationship/activity/interest, it is preferable that you do so.

The following questions apply to the author's relationships/activities/interests as they relate to the current manuscript only.

The author's relationships/activities/interests should be defined broadly. For example, if your manuscript pertains to the epidemiology of hypertension, you should declare all relationships with manufacturers of antihypertensive medication, even if that medication is not mentioned in the manuscript.

In item #1 below, report all support for the work reported in this manuscript without time limit. For all other items, the time frame for disclosure is the past 36 months.

		**Name all entities with whom you have this relationship or indicate none (add rows as needed)**	**Specifications/Comments (e.g., if payments were made to you or to your institution)**
		**Time frame: Since the initial planning of the work**	
1	All support for the present manuscript (e.g., funding, provision of study materials, medical writing, article processing charges, etc.) **No time limit for this item.**	**X** None	
		**Time frame: past 36 months**	
2	Grants or contracts from any entity (if not indicated in item #1 above).	____None	
3	Royalties or licenses	- None	

4	Consulting fees	____None	
5	Payment or honoraria for lectures, presentations, speakers bureaus, manuscript writing or educational events	____None	
6	Payment for expert testimony	**X** None	
7	Support for attending meetings and/or travel	____None	
8	Patents planned, issued or pending	**X** None	
9	Participation on a Data Safety Monitoring Board or Advisory Board	____None	
10	Leadership or fiduciary role in other board, society, committee or advocacy group, paid or unpaid	**X** None	
11	Stock or stock options	**X** None	
12	Receipt of equipment, materials, drugs, medical writing, gifts or other services	**X** None	
13	Other financial or non-financial interests	**X** None	

Please place an "X" next to the following statement to indicate your agreement:

X I certify that I have answered every question and have not altered the wording of any of the questions on this form.

ICMJE DISCLOSURE FORM

Date: 31 August 2022
Your Name: Mirella Fraquelli
Manuscript Title: CLINICAL AND GENETIC DETERMINANTS OF THE FATTY LIVER - COAGULATION BALANCE INTERPLAY IN INDIVIDUALS WITH METABOLIC DYSFUNCTION

Manuscript number (if known): HEPR-D-22-00290-R1

In the interest of transparency, we ask you to disclose all relationships/activities/interests listed below that are related to the content of your manuscript. "Related" means any relation with for-profit or not-for-profit third parties whose interests may be affected by the content of the manuscript. Disclosure represents a commitment to transparency and does not necessarily indicate a bias. If you are in doubt about whether to list a relationship/activity/interest, it is preferable that you do so.

The following questions apply to the author's relationships/activities/interests as they relate to the current manuscript only.

The author's relationships/activities/interests should be defined broadly. For example, if your manuscript pertains to the epidemiology of hypertension, you should declare all relationships with manufacturers of antihypertensive medication, even if that medication is not mentioned in the manuscript.

In item #1 below, report all support for the work reported in this manuscript without time limit. For all other items, the time frame for disclosure is the past 36 months.

		Name all entities with whom you have this relationship or indicate none (add rows as needed)	Specifications/Comments (e.g., if payments were made to you or to your institution)
		**Time frame: Since the initial planning of the work**	
1	All support for the present manuscript (e.g., funding, provision of study materials, medical writing, article processing charges, etc.) **No time limit for this item.**	**X** None	
		**Time frame: past 36 months**	
2	Grants or contracts from any entity (if not indicated in item #1 above).	____None	
3	Royalties or licenses	- None	

4	Consulting fees	____None	
5	Payment or honoraria for lectures, presentations, speakers bureaus, manuscript writing or educational events	____None	
6	Payment for expert testimony	**X** None	
7	Support for attending meetings and/or travel	____None	
8	Patents planned, issued or pending	**X** None	
9	Participation on a Data Safety Monitoring Board or Advisory Board	____None	
10	Leadership or fiduciary role in other board, society, committee or advocacy group, paid or unpaid	**X** None	
11	Stock or stock options	**X** None	
12	Receipt of equipment, materials, drugs, medical writing, gifts or other services	**X** None	
13	Other financial or non-financial interests	**X** None	

Please place an "X" next to the following statement to indicate your agreement:

X I certify that I have answered every question and have not altered the wording of any of the questions on this form.

ICMJE DISCLOSURE FORM

Date: 31 August 2022
Your Name: Daniele Prati
Manuscript Title: CLINICAL AND GENETIC DETERMINANTS OF THE FATTY LIVER - COAGULATION BALANCE INTERPLAY IN INDIVIDUALS WITH METABOLIC DYSFUNCTION

Manuscript number (if known): HEPR-D-22-00290-R1

In the interest of transparency, we ask you to disclose all relationships/activities/interests listed below that are related to the content of your manuscript. "Related" means any relation with for-profit or not-for-profit third parties whose interests may be affected by the content of the manuscript. Disclosure represents a commitment to transparency and does not necessarily indicate a bias. If you are in doubt about whether to list a relationship/activity/interest, it is preferable that you do so.

The following questions apply to the author's relationships/activities/interests as they relate to the current manuscript only.

The author's relationships/activities/interests should be defined broadly. For example, if your manuscript pertains to the epidemiology of hypertension, you should declare all relationships with manufacturers of antihypertensive medication, even if that medication is not mentioned in the manuscript.

In item #1 below, report all support for the work reported in this manuscript without time limit. For all other items, the time frame for disclosure is the past 36 months.

		**Name all entities with whom you have this relationship or indicate none (add rows as needed)**	**Specifications/Comments (e.g., if payments were made to you or to your institution)**
**Time frame: Since the initial planning of the work**			
1	All support for the present manuscript (e.g., funding, provision of study materials, medical writing, article processing charges, etc.) **No time limit for this item.**	**X** None	
**Time frame: past 36 months**			
2	Grants or contracts from any entity (if not indicated in item #1 above).	____None	
3	Royalties or licenses	- None	

4	Consulting fees	____None	
5	Payment or honoraria for lectures, presentations, speakers bureaus, manuscript writing or educational events	____None	Ortho Clinical Diagnostics, Grifols, Gilead, Terumo, Immucor, Diamed, Diasorin.
6	Payment for expert testimony	**X** None	
7	Support for attending meetings and/or travel	____None	Macopharma, Ortho Clinical Diagnostics, Grifols, Gilead, Terumo, Immucor, Diamed, Diasorin.
8	Patents planned, issued or pending	**X** None	
9	Participation on a Data Safety Monitoring Board or Advisory Board	____None	Macopharma
10	Leadership or fiduciary role in other board, society, committee or advocacy group, paid or unpaid	**X** None	
11	Stock or stock options	**X** None	
12	Receipt of equipment, materials, drugs, medical writing, gifts or other services	**X** None	
13	Other financial or non-financial interests	**X** None	

Please place an "X" next to the following statement to indicate your agreement:

X I certify that I have answered every question and have not altered the wording of any of the questions on this form.

ICMJE DISCLOSURE FORM

Date: 31 August 2022
Your Name: Vincenzo La Mura
Manuscript Title: CLINICAL AND GENETIC DETERMINANTS OF THE FATTY LIVER - COAGULATION BALANCE INTERPLAY IN INDIVIDUALS WITH METABOLIC DYSFUNCTION

Manuscript number (if known): HEPR-D-22-00290-R1

In the interest of transparency, we ask you to disclose all relationships/activities/interests listed below that are related to the content of your manuscript. "Related" means any relation with for-profit or not-for-profit third parties whose interests may be affected by the content of the manuscript. Disclosure represents a commitment to transparency and does not necessarily indicate a bias. If you are in doubt about whether to list a relationship/activity/interest, it is preferable that you do so.

The following questions apply to the author's relationships/activities/interests as they relate to the current manuscript only.

The author's relationships/activities/interests should be defined broadly. For example, if your manuscript pertains to the epidemiology of hypertension, you should declare all relationships with manufacturers of antihypertensive medication, even if that medication is not mentioned in the manuscript.

In item #1 below, report all support for the work reported in this manuscript without time limit. For all other items, the time frame for disclosure is the past 36 months.

		**Name all entities with whom you have this relationship or indicate none (add rows as needed)**	**Specifications/Comments (e.g., if payments were made to you or to your institution)**
		**Time frame: Since the initial planning of the work**	
1	All support for the present manuscript (e.g., funding, provision of study materials, medical writing, article processing charges, etc.) **No time limit for this item.**	**X** None	
		**Time frame: past 36 months**	
2	Grants or contracts from any entity (if not indicated in item #1 above).	____None	
3	Royalties or licenses	**-** None	

4	Consulting fees	____None	
5	Payment or honoraria for lectures, presentations, speakers bureaus, manuscript writing or educational events	Gore, Alfasigma, CSL Behring	Payment to me
6	Payment for expert testimony	Takeda, Biomarin	Payment to me
7	Support for attending meetings and/or travel	____None	
8	Patents planned, issued or pending	**X** None	
9	Participation on a Data Safety Monitoring Board or Advisory Board	____None	
10	Leadership or fiduciary role in other board, society, committee or advocacy group, paid or unpaid	**X** None	
11	Stock or stock options	**X** None	
12	Receipt of equipment, materials, drugs, medical writing, gifts or other services	**X** None	
13	Other financial or non-financial interests	**X** None	

Please place an "X" next to the following statement to indicate your agreement:

X I certify that I have answered every question and have not altered the wording of any of the questions on this form.

ICMJE DISCLOSURE FORM

Date: 31 August 2022
Your Name: Armando Tripodi
Manuscript Title: CLINICAL AND GENETIC DETERMINANTS OF THE FATTY LIVER - COAGULATION BALANCE INTERPLAY IN INDIVIDUALS WITH METABOLIC DYSFUNCTION

Manuscript number (if known): HEPR-D-22-00290-R1

In the interest of transparency, we ask you to disclose all relationships/activities/interests listed below that are related to the content of your manuscript. "Related" means any relation with for-profit or not-for-profit third parties whose interests may be affected by the content of the manuscript. Disclosure represents a commitment to transparency and does not necessarily indicate a bias. If you are in doubt about whether to list a relationship/activity/interest, it is preferable that you do so.

The following questions apply to the author's relationships/activities/interests as they relate to the current manuscript only.

The author's relationships/activities/interests should be defined broadly. For example, if your manuscript pertains to the epidemiology of hypertension, you should declare all relationships with manufacturers of antihypertensive medication, even if that medication is not mentioned in the manuscript.

In item #1 below, report all support for the work reported in this manuscript without time limit. For all other items, the time frame for disclosure is the past 36 months.

		**Name all entities with whom you have this relationship or indicate none (add rows as needed)**	**Specifications/Comments (e.g., if payments were made to you or to your institution)**
**Time frame: Since the initial planning of the work**			
1	All support for the present manuscript (e.g., funding, provision of study materials, medical writing, article processing charges, etc.) **No time limit for this item.**	**X** None	
**Time frame: past 36 months**			
2	Grants or contracts from any entity (if not indicated in item #1 above).	_X___None	
3	Royalties or licenses	- None	

4	Consulting fees	__X__None	
5	Payment or honoraria for lectures, presentations, speakers bureaus, manuscript writing or educational events	____None	
		Honoraria for lecture	Takeda, Roche, Stago, Werfen
6	Payment for expert testimony	**X** None	
7	Support for attending meetings and/or travel	X____None	
8	Patents planned, issued or pending	**X** None	
9	Participation on a Data Safety Monitoring Board or Advisory Board	X___None	
10	Leadership or fiduciary role in other board, society, committee or advocacy group, paid or unpaid	**X** None	
11	Stock or stock options	**X** None	
12	Receipt of equipment, materials, drugs, medical writing, gifts or other services	**X** None	
13	Other financial or non-financial interests	**X** None	

Please place an "X" next to the following statement to indicate your agreement:

X I certify that I have answered every question and have not altered the wording of any of the questions on this form.

ICMJE DISCLOSURE FORM

Date: 31 August 2022
Your Name: Flora Peyvandi
Manuscript Title: CLINICAL AND GENETIC DETERMINANTS OF THE FATTY LIVER - COAGULATION BALANCE INTERPLAY IN INDIVIDUALS WITH METABOLIC DYSFUNCTION

Manuscript number (if known): HEPR-D-22-00290-R1

In the interest of transparency, we ask you to disclose all relationships/activities/interests listed below that are related to the content of your manuscript. "Related" means any relation with for-profit or not-for-profit third parties whose interests may be affected by the content of the manuscript. Disclosure represents a commitment to transparency and does not necessarily indicate a bias. If you are in doubt about whether to list a relationship/activity/interest, it is preferable that you do so.

The following questions apply to the author's relationships/activities/interests as they relate to the current manuscript only.

The author's relationships/activities/interests should be defined broadly. For example, if your manuscript pertains to the epidemiology of hypertension, you should declare all relationships with manufacturers of antihypertensive medication, even if that medication is not mentioned in the manuscript.

In item #1 below, report all support for the work reported in this manuscript without time limit. For all other items, the time frame for disclosure is the past 36 months.

		**Name all entities with whom you have this relationship or indicate none (add rows as needed)**	**Specifications/Comments (e.g., if payments were made to you or to your institution)**
**Time frame: Since the initial planning of the work**			
1	All support for the present manuscript (e.g., funding, provision of study materials, medical writing, article processing charges, etc.) **No time limit for this item.**	**X** None	
**Time frame: past 36 months**			
2	Grants or contracts from any entity (if not indicated in item #1 above).	____None	
3	Royalties or licenses	- None	

4	Consulting fees	____None	
5	Payment or honoraria for lectures, presentations, speakers bureaus, manuscript writing or educational events	Grifols, Roche	To me
6	Payment for expert testimony	**X** None	
7	Support for attending meetings and/or travel	____None	
8	Patents planned, issued or pending	**X** None	
9	Participation on a Data Safety Monitoring Board or Advisory Board	Sanofi, Sobi, Takeda, Roche, Biomarin	To me
10	Leadership or fiduciary role in other board, society, committee or advocacy group, paid or unpaid	**X** None	
11	Stock or stock options	**X** None	
12	Receipt of equipment, materials, drugs, medical writing, gifts or other services	**X** None	
13	Other financial or non-financial interests	**X** None	

Please place an "X" next to the following statement to indicate your agreement:

X I certify that I have answered every question and have not altered the wording of any of the questions on this form.
